# Synergistic action of the transcription factors Krüppel homolog 1 and Hairy in juvenile hormone/Methoprene-tolerant-mediated gene-repression in the mosquito *Aedes aegypti*

**DOI:** 10.1371/journal.pgen.1008443

**Published:** 2019-10-29

**Authors:** Tusar T. Saha, Sourav Roy, Gaofeng Pei, Wei Dou, Zhen Zou, Alexander S. Raikhel

**Affiliations:** 1 Department of Entomology and Institute of Integrative Biology, University of California, Riverside, California, United States of America; 2 Department of Biological Sciences, Birla Institute of Technology and Science Pilani, K. K. Birla Goa Campus, Goa, India; 3 Department of Biological Sciences, University of Texas El Paso, Texas; 4 State Key Laboratory of Integrated Management of Pest Insects and Rodents, Institute of Zoology, Chinese Academy of Sciences, Beijing, China; 5 University of Chinese Academy of Sciences, Beijing, China; 6 College of Plant Protection, Southwest University, Chongqing, China; Howard Hughes Medical Institute, UNITED STATES

## Abstract

Arthropod-specific juvenile hormones control numerous essential functions in development and reproduction. In the dengue-fever mosquito *Aedes aegypti*, in addition to its role in immature stages, juvenile hormone III (JH) governs post-eclosion (PE) development in adult females, a phase required for competence acquisition for blood feeding and subsequent egg maturation. During PE, JH through its receptor Methoprene-tolerant (Met) regulate the expression of many genes, causing either activation or repression. Met-mediated gene repression is indirect, requiring involvement of intermediate repressors. Hairy, which functions downstream of Met in the JH gene-repression hierarchy, is one such factor. Krüppel-homolog 1, a zinc-finger transcriptional factor, is directly regulated by Met and has been implicated in both activation and repression of JH-regulated genes. However, the interaction between Hairy and Kr-h1 in the JH-repression hierarchy is not well understood. Our RNAseq-based transcriptomic analysis of the Kr-h1-depleted mosquito fat body revealed that 92% of Kr-h1 repressed genes are also repressed by Met, supporting the existence of a hierarchy between Met and Kr-h1 as previously demonstrated in various insects. Notably, 130 genes are co-repressed by both Kr-h1 and Hairy, indicating regulatory complexity of the JH-mediated PE gene repression. A mosquito Kr-h1 binding site in genes co-regulated by this factor and Hairy was identified computationally. Moreover, this was validated using electrophoretic mobility shift assays. A complete phenocopy of the effect of Met RNAi depletion on target genes could only be observed after Kr-h1 and Hairy double RNAi knockdown, suggesting a synergistic action between these two factors in target gene repression. This was confirmed using a cell-culture-based luciferase reporter assay. Taken together, our results indicate that Hairy and Kr-h1 not only function as intermediate downstream factors, but also act together in a synergistic fashion in the JH/Met gene repression hierarchy.

## Author summary

Juvenile hormone (JH) plays an essential role in preparing *Aedes aegypti* female mosquitoes for blood feeding, egg development, and pathogen transmission. JH acting through its receptor Methoprene-tolerant (Met) regulates the expression of large gene cohorts. JH mediated gene repression, unlike activation that is directly mediated by Met, is indirect and requires intermediate transcriptional repressors Hairy and Krüppel-homolog 1 (Kr-h1). Here, we demonstrate that Hairy and Kr-h1 can act synergistically in the JH-Met gene repression pathway in *Aedes* female mosquitoes. These interact directly with regulatory regions of the genes that have both Hairy and Kr-h1 binding sites. Thus, this study has significantly advanced our understanding of the complexity of the JH-mediated gene expression pathway. This research yields valuable information about the JH control of reproductive development of the mosquito *A*. *aegypti*, one of the most important vectors of human diseases.

## Introduction

Arthropod-specific juvenile hormones (JHs) are key regulators of a large array of physiological processes, including growth, metamorphosis and reproduction [1, 2]. Methoprene-tolerant (Met), a basic helix-loop-helix (bHLH)/-Per-Arnt-Sim (PAS) domain protein, has been characterized as the insect JH receptor [3–8]. Additionally, Taiman (Tai), an insect homolog of the vertebrate steroid receptor co-activator (SRC), which is also a bHLH-PAS protein, has been identified as the Met obligatory partner [9, 10]. The heterodimer formation of Met/Tai, necessary for successful transduction of the JH signal, is induced by the ligand-receptor (JH-Met) interaction [11]. In the nucleus, the JH receptor complex interacts with JH response elements (JHREs), usually containing the core E-box-like motif ‘CACGTG’, in regulatory regions of target genes leading to their activation [9, 12–17]. Details of the nuclear import of Met have also been investigated, revealing the identity of Met-interacting chaperone heat shock protein 83 (Hsp83), which in turn works together with the components of the nuclear pore complex facilitating Met nuclear translocation [18, 19]. Additionally, recent studies have indicated the existence of a not-yet fully characterized membrane JH receptor that is responsible for Met phosphorylation, a necessary step for the activity of the JH receptor complex [20, 21]. This mode of action involving JH has been confirmed for several genes, including *early trypsin* (*ET*), *regulator of ribosomal synthesis 1* (*RRS1*), *Hairy*, and *Krüppel-homolog 1* (*Kr-h1*) [2, 10, 12, 16, 17, 22, 23]. Some targets of the JH signaling pathway, such as Hairy and Kr-h1, are transcription factors (TFs) that in turn can regulate the expression of downstream genes [2, 22, 23].

In the mosquito *A*. *aegypti*, JH III (JH) is the principal hormone controlling the physiological maturation in newly eclosed adult females. The JH-governed developmental period spans from adult eclosion to blood feeding, termed post-eclosion (PE) phase, and is critical for subsequent reproductive maturation and successful egg production. The JH titer increases after adult eclosion, reaching a peak at 48-54h PE [24]. The mosquito PE phase is characterized by sequential waves of highly expressed fat body (FB) genes, which are modulated by differential JH titers [15]. Met plays a central role in JH-mediated PE gene expression, regulating a total of 2151 transcripts, of which 1613 are upregulated and 538 downregulated [22]. A bioinformatics approach has predicted that, unlike activation, Met-mediated gene repression is indirect and requires the involvement of additional downstream factors [15]. Indeed, we have identified the bHLH-Orange domain protein Hairy as an intermediate factor in JH/Met-mediated gene repression [22]. Hairy binds to target gene promoters (the E-box sequence or its variants) and recruits the co-repressor Groucho (Gro1), resulting in transcriptional repression of JH/Met/Hairy target genes [22]. *Hairy* is a late PE gene, having a low expression level in newly eclosed mosquito FB, gradually increasing to reach a peak at around 60h PE and maintaining high expression levels throughout rest of the PE phase [15, 22]. Gro1, one the other hand, is constitutively expressed throughout PE indicating that it is the recruitment of the protein, and not its availability, that plays a crucial role in Hairy-mediated gene repression downstream of JH/Met [15, 22].

Another intermediate factor that has been implicated in JH/Met gene repression is the C_2_H_2_ zinc-finger TF Kr-h1 [25]. *Kr-h1* has been characterized as an early inducible gene in the JH signaling pathway downstream of Met in *Drosophila* and *Tribolium castaneum* [26, 27]. The JH-receptor complex directly induces *Kr-h1* expression by interacting with JH response elements in the upstream regulatory region [8, 9, 11–14, 16]. Kr-h1 has been demonstrated to repress two key genes, Broad-complex (BR-C) and E93, during JH-regulated metamorphosis in various insects [26–31]. In adult female *A*. *aegypti* mosquitoes, *Kr-h1* shows an expression pattern similar to that of *Hairy* and is characterized as a late PE gene [15]. As in the case for Hairy, mosquito Kr-h1 also functions downstream of Met in the JH-mediated gene-repression hierarchy [23]. Whether Kr-h1 and Hairy interact in this hierarchy is, however, not understood. Our comparative analysis of RNA interference (RNAi) PE fat body transcriptomes for *Kr-h1* and *Hairy* has revealed a significant overlap, suggesting simultaneous involvement of these two Met-regulated factors in gene repression. Only a simultaneous RNAi knockdown of *Hairy* and *Kr-h1* could fully phenocopy the effect of Met depletion. Our results have suggested a synergistic action of Kr-h1 and Hairy in JH/Met-mediated gene repression, a hypothesis that was verified using cell transfection assays.

Our findings provide an important mechanistic insight into the JH signaling pathway. JH is a structurally unique and arthropod-specific hormone, the chemical analogs of which have been routinely utilized as potent insecticides. Furthermore, JH is a crucial component of the mosquito reproductive cycle, which in turn forms the basis for acquisition and transmission of pathogens of such devastating diseases as malaria, dengue, chikungunya, yellow fever and zika virus. Thus, these findings might lead to the identification of potential novel targets for mosquito control.

## Results

### Transcriptomic analysis of Kr-h1 depleted mosquito FB tissue

**Fig 1:**
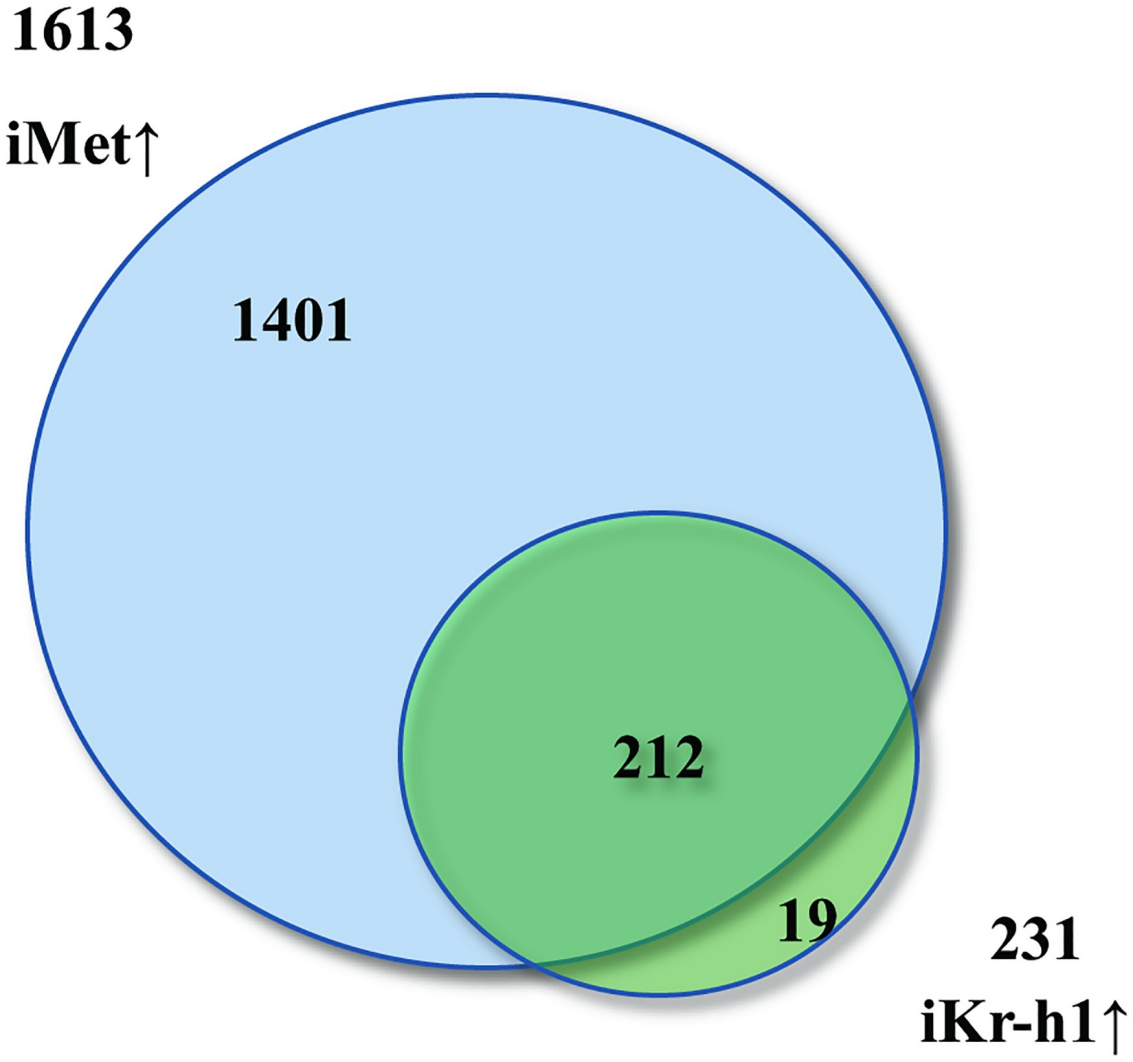
Transcriptomic analysis of Kr-h1 depleted mosquito FB. Illumina RNA-seq based transcriptomic analysis of *Kr-h1* RNAi-depleted (*iKr-h1*) female *A*. *aegypti* fat body. RNAi-depleted Luciferase (*iLuc*) fat body transcriptome was used as a control. Venn diagram comparing *iMet* and *iKr-h1* transcriptomes shows that 92% of RNAi Kr-h1 (iKr-h1) upregulated genes (212 genes) were also upregulated by *iMet*. *iMet***↑**, indicates more than two-fold upregulation by dsRNA-mediated depletion of *Met*; *iKr-h1***↑**, more than two-fold upregulation by dsRNA-mediated depletion of *Kr-h1*.

To investigate the role of Kr-h1 in JH/Met-mediated gene repression, we performed an RNAi-based transcriptomic screen for this TF. Illumina RNA-seq technology was used to identify genes regulated by dsRNA-mediated knockdown of *Kr-h1* (iKr-h1) in the female mosquito FB. The high-throughput experiments were performed as previously described [22]. Setting a cut-off log-fold change of more than +1 (2-fold change), a total of 231 transcripts were found to be upregulated in *Kr-h1* RNAi-depleted mosquitoes when compared with the RNAi Luciferase (iLuc) control (S1 Dataset). Of the transcripts activated by *Kr-h1* RNAi, 92% were also activated by the RNAi mediated knockdown of *Met* (Fig 1, S1 Dataset).

To verify the reproducibility of the result, two additional biological replicates of RNA-seq libraries were constructed for each RNAi treatment followed by sequencing. Results like that mentioned above were also obtained for the two repeats, with activation of 244 and 223 transcripts in *iKr-h1* samples of replicates 2 and 3, respectively (S2 Dataset). Of the *iKr-h1*↑ transcripts from replicates 2 and 3, 91% and 90%, respectively, were also induced by the knockdown of *Met* (S2 Dataset). Comparison of transcripts activated by *iKr-h1* in the three biological replicates revealed that 207 transcripts were common to all three samples (S1 Fig), indicating reproducibility of the parallel experiments.

The overlap between *Met* and *Kr-h1* RNAi-depleted transcriptomes suggests a hierarchy in the JH-mediated repression of target genes. Kr-h1 has been implicated to play a role as an intermediate factor downstream of Met in gene repression by JH in various insects [26–31], including mosquitoes [23]. Our results agree with these data.

### Analysis of genes co-repressed by both Hairy and Kr-h1

**Fig 2:**
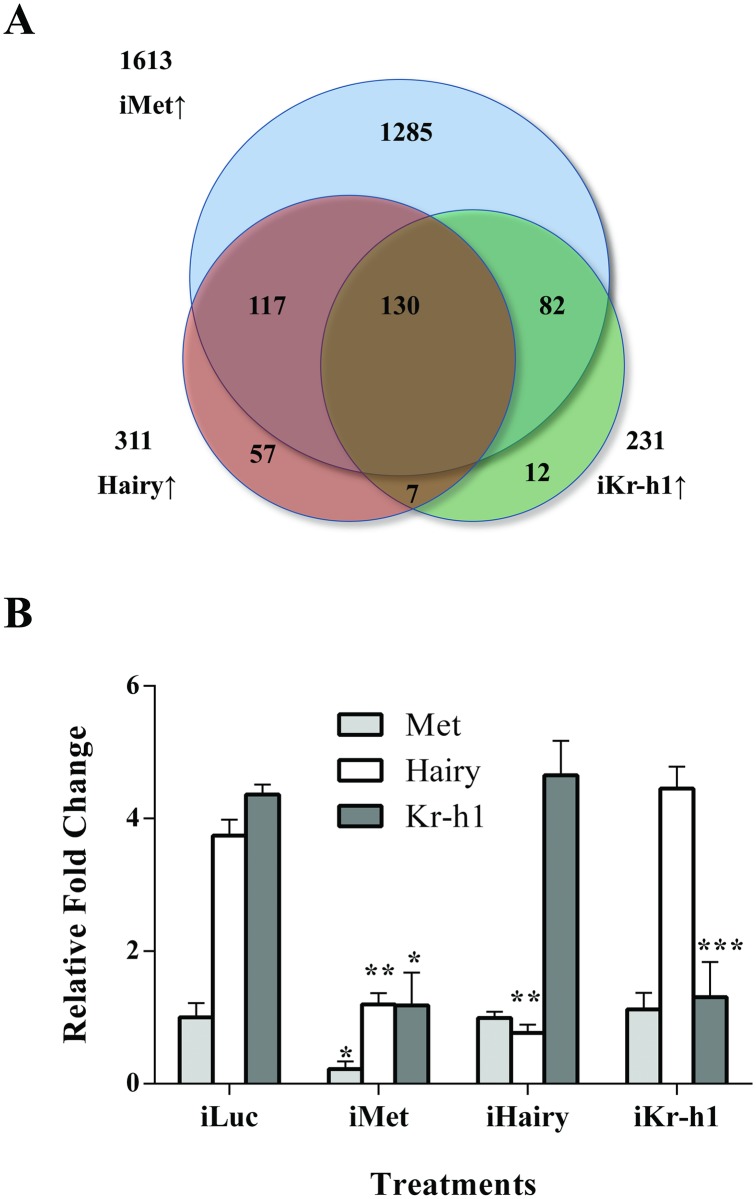
Comparative analysis of *iKr-h1* and *iHairy* transcriptomes shows a significant overlap between these two gene sets. **(A)** Venn diagram comparing *iMet*, *iHairy* and *iKr-h1* transcriptomes revealed that 130 target genes were activated by both *iHairy* and *iKr-h1* in the *iMet* background, indicating that the two factors acted either co-operatively or hierarchically in repression of JH/Met target genes. *iMet***↑**, more than two-fold upregulation by dsRNA depletion of Met; *iKr-h1***↑**, more than two-fold upregulation by dsRNA-mediated depletion of Kr-h1; *iHairy***↑**, more than two-fold upregulation by dsRNA-mediated depletion of Hairy. **(B)** qRT-PCR analysis of Met, Kr-h1 and Hairy mRNA expression in *iMet*, *iHairy* and *iKr-h1* mosquito fat body. iLuc was used as control. The knockdown of Hairy did not impact the expression of Kr-h1 mRNA or vice versa, ruling out a hierarchy between the two factors. Error bars represent ± SD. *p < 0.05; **p < 0.01; ***p < 0.001.

It is clear from previous publications and our present results that Kr-h1 and Hairy are two TFs that act downstream of Met in the JH gene-repression hierarchy [22, 23]. We identified a large overlap (137 transcripts) between the *Kr-h1*- and *Hairy* RNAi-upregulated transcriptomes (Fig 2A and S3 Dataset). Of the *iHairy* and *iKr-h1* upregulated genes, 44.1% (137/311) and 59.3% (137/231), respectively, were found to be common among the transcripts regulated by the two TFs. Of these 137 transcripts, 130 were also found to be repressed by Met (Fig 2A and S3 Dataset). Similar results were also observed in the other two biological replicates.

**Fig 3:**
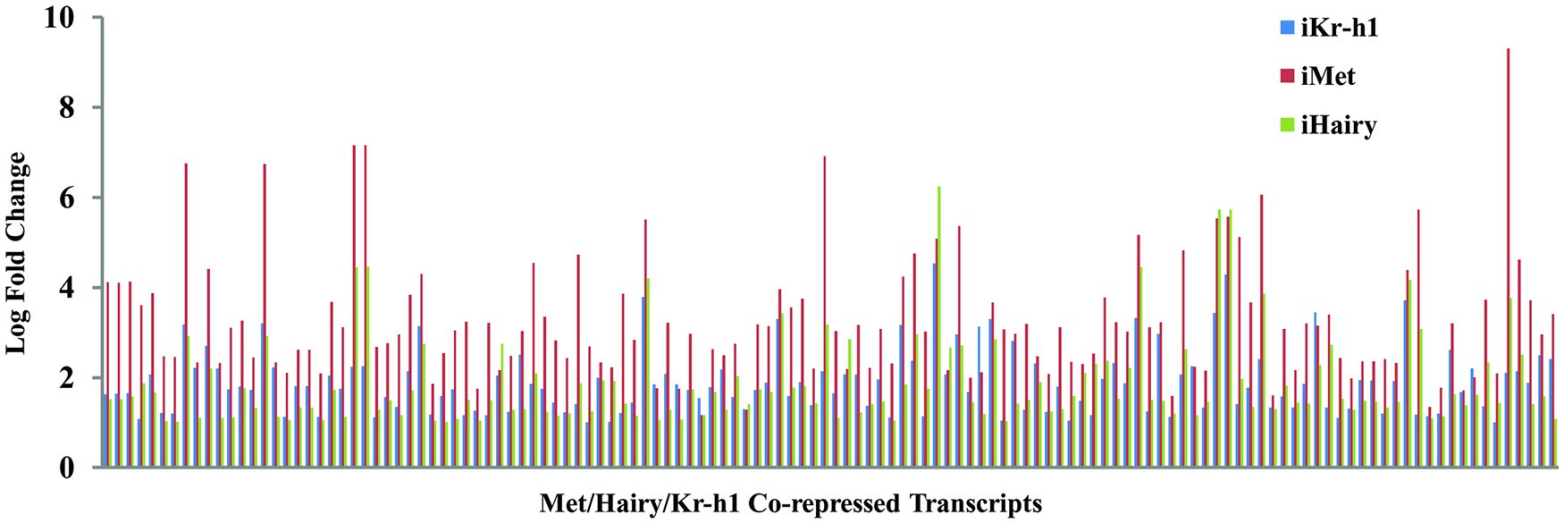
Differences in the expression intensities of Met/Hairy/Kr-h1 co-repressed genes among *iMet*, *iHairy* and *iKr-h1* samples. Expression intensities of the 130 genes activated by RNAi-mediated knockdowns of *Met*, *Hairy* and *Kr-h1* as revealed by RNA-seq analysis. The expression intensities are represented on a Log_2_ scale. Systemic differences among the *iMet*, *iHairy* and *iKr-h1* samples are observed, with *iMet* knockdowns resulting in higher induction in the expression of target transcripts in comparison to either *iHairy* or *iKr-h1*.

qRT-PCR analysis of the expression of *Met*, *Hairy* and *Kr-h1* in *iMet*, *iHairy* and *iKr-h1* samples suggests that neither Hairy nor Kr-h1 regulates the transcription of each other, ruling out a hierarchy between these two factors (Fig 2B). Therefore, the observation that a significant number of genes downstream of Met are regulated by both Hairy and Kr-h1 led us to the hypothesis that Hairy and Kr-h1 act synergistically in JH/Met-regulated gene repression. A careful look at the fold changes of the target genes indicated widespread differences among the *iMet*, *iHairy* and *iKr-h1* transcriptomes (Fig 3). In most of these Met/Hairy/Kr-h1 repressed genes, the intensity of activation as a result of *Met* knockdown was significantly higher than that in either *iHairy* or *iKr-h1* treatments (Fig 3). The trend was also observed in the other two biological repeats.

**Fig 4:**
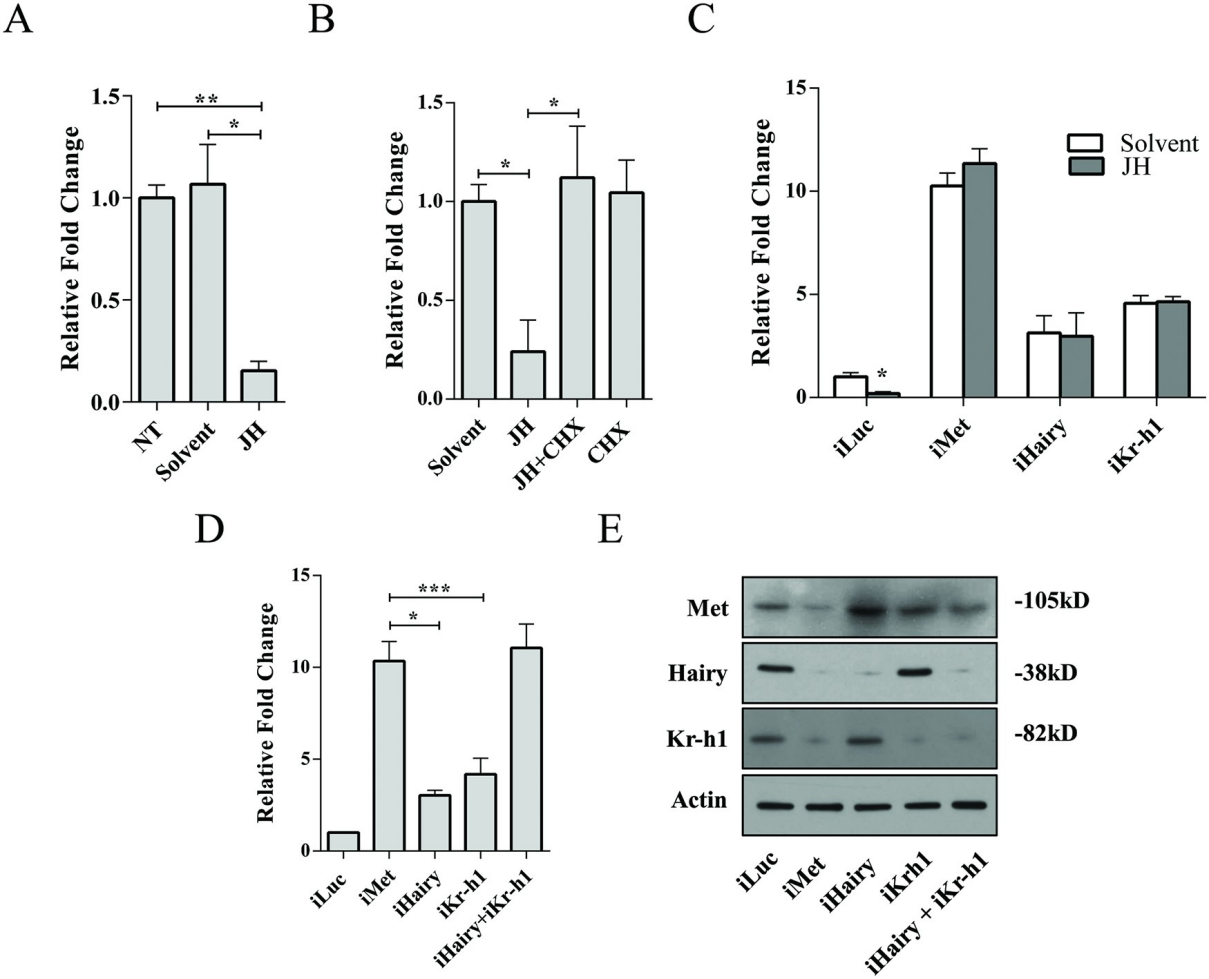
Simultaneous knockdown of *Kr-h1* and *Hairy* phenocopies the effects of *Met* RNAi depletion on the expression of the Met/Hairy/Kr-h1 target gene. **(A)** Hormonal application experiments showing the effect of JH on the expression of *AAEL005093 (Clip-Domain Serine Protease)*. A clear repression of the tested gene was observed in JH-treated samples. NT, no treatment. **(B)** The necessity of intermediate factors for the JH-mediated repression of the target gene *AAEL005093* as determined by *in-vitro* fat body culture experiments. JH-mediated repression was compromised by the addition of CHX into the tissue culture medium (JH+CHX). Solvent (acetone) and CHX-treated samples were used as controls. **(C)** *In-vitro* fat body culture experiments demonstrating the effect of JH on the expression of *AAEL005093* in *iLuc*, *iMet*, *iHairy* and *iKr-h1* samples. **(D)** qRT-PCR-based expression analysis of Met/Hairy/Kr-h1 target gene *AAEL005093* in *iMet*, *iHairy*, *iKr-h1* and *iHairy*+*iKr-h1* samples. *iLuc* samples were used as controls. **(E)** Western blot analysis, showing the protein levels of Met, Hairy and Kr-h1 in the samples of the above experiment (4D), indicates the efficiency and specificity of RNAi knockdowns. Error bars represent ± SD. *p < 0.05; ***p < 0.001.

We conducted further experiments to establish JH repression of the Met/Hairy/Kr-h1-regulated gene *AAEL005093* (*Clip-Domain Serine Protease*). We first examined the effect of JH by treating newly eclosed female mosquitoes with JH III. qRT-PCR analyses revealed a clear repression of the tested gene *AAEL005093* by JH in comparison with the acetone-treated (Solvent) or untreated (NT) control mosquitoes (Fig 4A). Next, we utilized cycloheximide (CHX), a potent translational blocker, in the *in-vitro* fat body culture (Solvent, JH, JH+CHX, CHX) according to Saha et al. (2016) to test the necessity of intermediate factors in the JH-mediated repression [22]. The tested gene transcript level was high after solvent (acetone) treatment but was significantly reduced in the JH-treated samples (Fig 4B). Addition of CHX to the JH-containing culture medium rendered the *AAEL005093* gene unresponsive to the repressive action of this hormone (Fig 4B), clearly indicating the involvement of intermediate factor(s) downstream of Met in regulation of target genes. To investigate the involvement of specific Met downstream factors, we performed experiments combining dsRNA-mediated knockdown and *in-vitro* fat body culture, as previously described [22]. FBs from mosquitoes treated with dsRNA for *Luc* (control), *Met*, *Hairy* or *Kr-h1* were incubated in the culture medium in the presence or absence of JH. As for iMet, knockdown of both Kr-h1 and Hairy resulted in a compromised JH action on the repression of the target gene *AAEL005093* (Fig 4C), establishing that both factors are involved in the JH-mediated regulation of the target gene.

To probe the synergistic action between Kr-h1 and Hairy, we performed simultaneous knockdown of the two factors (Fig 4D). The expression of *AAEL005093* was induced in all the test samples—*iMet*, *iKr-h1*, *iHairy* and *iKr-h1*+*iHairy* in comparison with *iLuc* controls. However, the intensity of induction of *AAEL005093* in *iKr-h1* and *iHairy* samples was significantly lower than in *iMet* samples (Fig 4D). However, the level of the target gene induction was comparable between *iMet* and *iHairy*+*iKr-h1* samples, suggesting a possible synergistic action of Hairy and Kr-h1 factors downstream of Met in the JH signaling pathway (Fig 4D). Specifically, the expression of *AAEL005093* in *iKr-h1*+*iHairy* samples was approximately 110% of that of *iMet*, 300% of *iHairy* and 400% of *iKr-h1* (Fig 4D). Efficiency and specificity of RNAi knockdowns in this experiment were confirmed using Western blot analysis (Fig 4E). We further confirmed our results by testing a second gene (*AAEL006978*, *protein-glutamine gamma-glutamyltransferase*) from this gene set. Like *AAEL005093*, *AAEL006978* is regulated by JH and requires intermediate factor downstream of Met for its repression (S2A and S2B Fig). Most importantly, *AAEL006978* expression is impacted to comparable levels in *iMet* and *iHairy*+*iKr-h1* samples, suggesting a synergistic action of these two factors in this gene as well (S2C Fig). The results of RNAi experiment for the two tested genes *AAEL005093* and *AAEL006978* conform to our RNAseq results (Fig 3 and S3 Dataset).

### Ontology analysis of genes co-repressed by Kr-h1, Hairy and Met

Gene ontology analysis based on adjusted non-supervised orthologous groups (NOGs) for the 130 genes co-repressed by Kr-h1 and Hairy in a Met background was performed as described previously [22]. The genes were mapped to two major functional categories—Cellular Processes and Signaling, and Metabolism—and none was mapped to the third major functional category—Information Storage and Processing. Significantly overrepresented OGs (*p*-value < 0.01 in a hypergeometric distribution) within Cellular Processes and Signaling are functional groups [O] posttranslational modification, protein turnover, chaperons, [V] defense mechanism and [T] signal transduction were well represented in this gene set (S3 Fig and S1 Table). Additionally, two transcripts were mapped to [W] extracellular processes under this broad category. Within Metabolism, the overrepresented groups are [P] inorganic ion transport and metabolism, and [Q] secondary metabolites biosynthesis, transport and catabolism (S3 Fig and S1 Table).

### Characterization of the *A*. *aegypti* Kr-h1 binding site

C_2_H_2_ domain containing Kr-h1 is expected to bind to specific DNA elements. Kayukawa et al. (2016) have demonstrated that Kr-h1 binds directly to a Kr-h1 binding site (KBS) in the 5′ regulatory region of the Broad complex (*BR-C*) gene in silkworm *Bombyx mori* [30]. In that study, the authors defined a 30-bp sequence—5′ GACCTACGCTAACGCTAAATAGAGTTCCGA 3′ —as the KBS core region. A more detailed EMSA study revealed that this 30-mer KBS core region binds two *Bombyx* Kr-h1 protein molecules, indicating that it might harbor two closely placed Kr-h1 binding sites [30]. It was also suggested that each zinc finger domain of *Bm*Kr-h1 recognizes a different sequence in the KBS core region, due to the diversity of the amino acid residues of the α helix of each zinc finger domain in *Bm*Kr-h1 [30]. In a subsequent study, the same group characterized KBS consensus using chromatin immunoprecipitation sequencing analysis (ChIP-seq) [32]. When aligned with KBS sequences in the promoters of *Bm*Br-C and *Bm*E93A, the identified KBS consensus demonstrated that 13 nucleotides of the 15-bp-long consensus sequence were highly conserved. Also, the first 9 conserved bases were separated from the last 4 by a more degenerate base. With the knowledge that the 30-mer KBS core region binds two *Bombyx* Kr-h1 protein molecules in the promoter of *Bm*BR-C and that each zinc finger domain of *Bm*Kr-h1 might recognize a different sequence in the KBS core region [30], we wanted to identify the Kr-h1 binding site in *A*. *aegypti* (*Aa*KBS). In particular, we were interested in detecting the *Aa*KBS within the upstream regions of the Met/Kr-h1/Hairy regulated genes.

**Fig 5:**
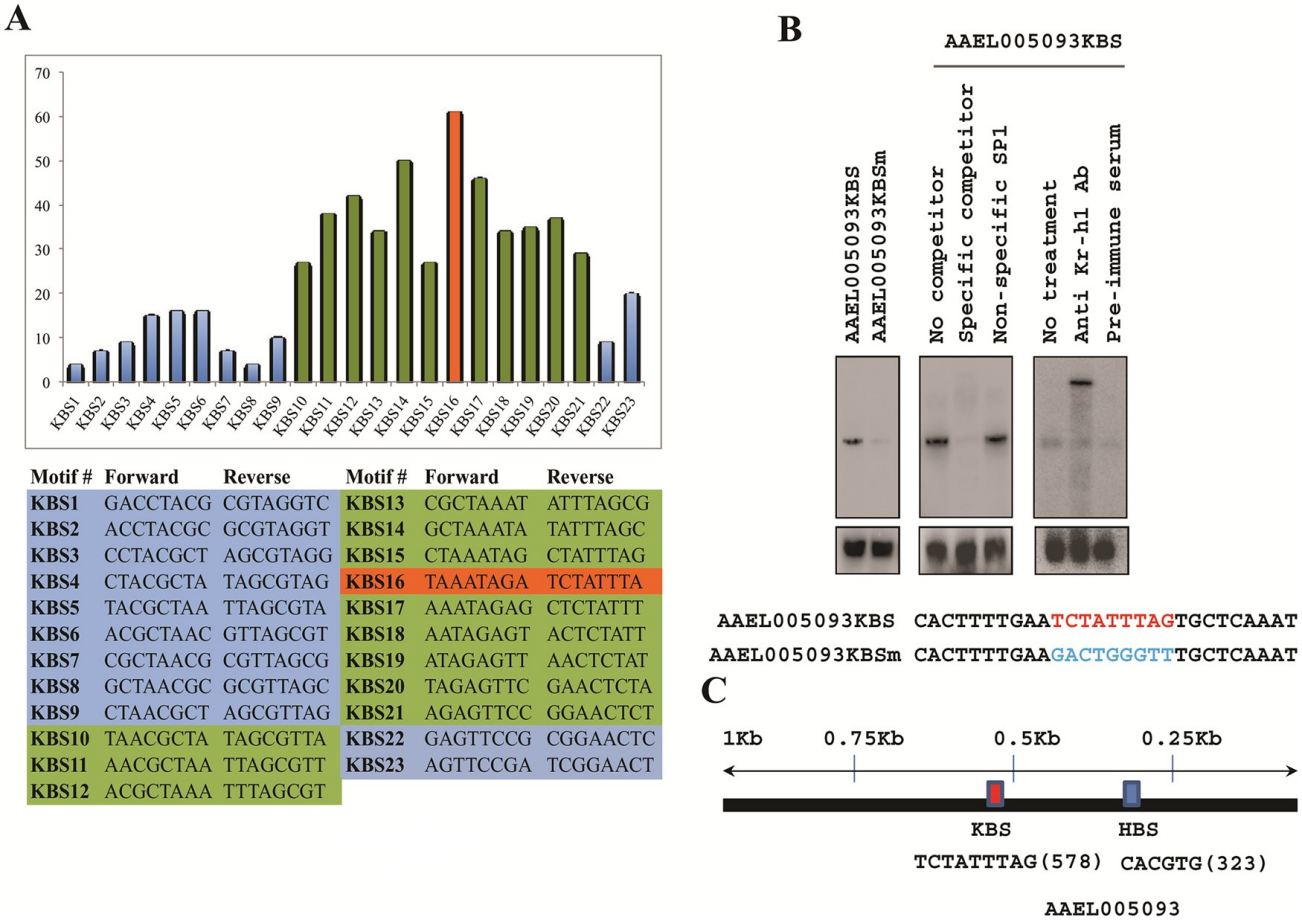
Characterization of *Aa*Kr-h1 interacting DNA motifs. **(A)** Bioinformatics analysis of the iKr-h1 transcriptome for the characterization of Kr-h1 binding site (KBS). The 30-mer *Bombyx mori* Kr-h1 binding site (5´GACCTACGCTAACGCTAAATAGAGTTCCGA3´) was used to search for 8-mer sequences with a one-base sliding window. The frequency of hits for all the search sequences in the region 5-kb upstream of *iMet*/*iKr-h1* upregulated transcripts is plotted here. In total, 23 search sequences were generated and are listed at the bottom. **(B)** EMSAs with fat-body nuclear extract (NE) from female *A*. *aegypti* 48 h PE and the putative *Aa*Kr-h1 binding site (KBS) ‘TCTATTTAG’ and its flanking regions from the promoter of Met/Hairy/Kr-h1 target gene *AAEL005093*. Mutation of the KBS to ‘GACTGGGTT’ abolished DNA-protein interaction. The specificity of the binding was confirmed by competition with unlabeled specific probe; non-specific SP1 (Promega) was used as control. The presence of Kr-h1 in the DNA–protein complex was verified by a super-shift with anti-*Aa*Kr-h1 polyclonal antibody; pre-immune serum was added as control. Lower panels show unbound probe as loading control. The motifs along with corresponding flanking sequences used for the EMSA assays are mentioned below. **(C)** Position of the KBS and HBS with respect to the TSS, located within 1kb upstream of the *AAEL005093* gene.

First, we analyzed 5-kb 5´ upstream regions of the Met- and Kr-h1-repressed genes. We used an 8-base sliding window to search 5-kb upstream regions of the upregulated transcripts that were activated after the depletion of both Met and Kr-h1. These regions were scanned for 23 different 8-mers from within the 30-mer KBS and their reverse complementary sequences, and the frequency of their occurrences (hits) was plotted (Fig 5A). The decision to divide the 30-mer KBS into 8-mers was based on the conservation demonstrated in the alignment between the *Bm*Kr-h1 ChIP-seq consensus and KBS sequences on the promoters of *BmE93A* and *BmBR-C* [32]. It was observed that for 12 (KBS10 -KBS21) out of the 23 different 8-mers, the number of putative occurrences within the dataset of 5´ upstream regions was statistically significant, that is the observed values were significantly higher than the values expected by random chance. Motif “TAAATAGA” (KBS16) and its reverse complementary sequence “TCTATTTA” scored the maximum number (61) of hits, suggesting that this sequence may be enough for binding Kr-h1 (Fig 5A).

Next, we checked regions 5-kb upstream of the Met/Kr-h1/Hairy downregulated genes in a similar way to that described above. It was observed that the number of hits for the same 8-mers (KBS10-KBS21) were statistically significant (S4 Fig). This suggested that *Aa*Kr-h1 may bind to any of these 12 KBSs or a combination of these putative sites depending on the actual length of the binding site.

To test *A*. *aegypti* Kr-h1 binding to the characterized KBS site, we used oligonucleotide sequences from the promoter region of a Met/Kr-h1/Hairy-repressed gene, AAEL005093, which carries the reverse complementary sequence ‘TCTATTTA’ of KBS16 that was shown to record the maximum number of hits (Fig 5A) in our bioinformatics analysis. We selected this promoter for EMSA analysis because most of the sequences on the right and left flanking regions of this core putative KBS did not match the 30-mer reported by Kayukawa et al. (2016) [30], other than a guanine “G” which is to the right of the last adenine “A”, essentially making a 9-mer core KBS that is a combination of KBS16 and KBS15. This presumed core *Aa*KBS ‘TCTATTTAG’ along with 10-bp flanks at both 3´ and 5´ ends of the KBS were then used for EMSA. We utilized *A*. *aegypti* fat body nuclear extracts, collected at 72 h PE, to perform these studies. Using the above-mentioned oligonucleotide harboring *Aa*KBS, a strong binding was observed, as evident from the distinct band in the EMSA assay (Fig 5B). Mutation of the *Aa*KBS ‘TCTATTTAG’ to ‘GACTGGGTT’, a sequence not predicted to bind Kr-h1 and very different from the consensus *Aa*KBS, resulted in complete disappearance of the band, indicating the absolute necessity of the KBS for the DNA-protein interaction. Competition assays with 50-fold excess of unlabeled specific probe, but not the nonspecific competitor SP1, resulted in the elimination of the observed band, thus indicating the specificity of the DNA-protein interaction (Fig 5B). Next, we investigated the presence of *A*. *aegypti* Kr-h1 protein in the DNA-protein complex by utilizing our custom-generated polyclonal anti-Kr-h1 antibody. Inclusion of this antibody in the EMSA reaction resulted in a super-shift of the band, indicating the presence of Kr-h1 protein in the DNA binding complex (Fig 5B).

Thus, we have identified a 9-mer sequence from the upstream regulatory region of an *A*. *aegypti* Met/Kr-h1/Hairy target gene that interacts with Kr-h1 protein. The core binding sequence was predicted as a KBS in our bioinformatics search, and it is expected that other variations of this KBS would also interact with Kr-h1. Validations of all these putative KBS sites are beyond the scope of this investigation.

### Distance correlation between Kr-h1 and Hairy binding sites in target gene promoters

We performed a bioinformatics analysis for the KBS and the Hairy Binding Sites (HBS, characterized in Saha et al. 2016 [22]) within the upstream regions of the Met/Hairy/Kr-h1-repressed genes to check whether we could detect any distance correlation between these two sites. In 92% of the genes analyzed, the distance between the two sites was less than 1kb. Interestingly, the two sites were separated by fewer than 300 bp in 70% of the genes (S2 Table). There was no clear orientation bias for the sites, as HBS was downstream of KBS in 53% of the genes and KBS was downstream of HBS in 47% (S2 Table). These results further support our hypothesis regarding synergistic action of Kr-h1 and Hairy in JH-mediated repression of target genes.

### Synergistic action of Kr-h1 and Hairy in the repression of target genes

**Fig 6:**
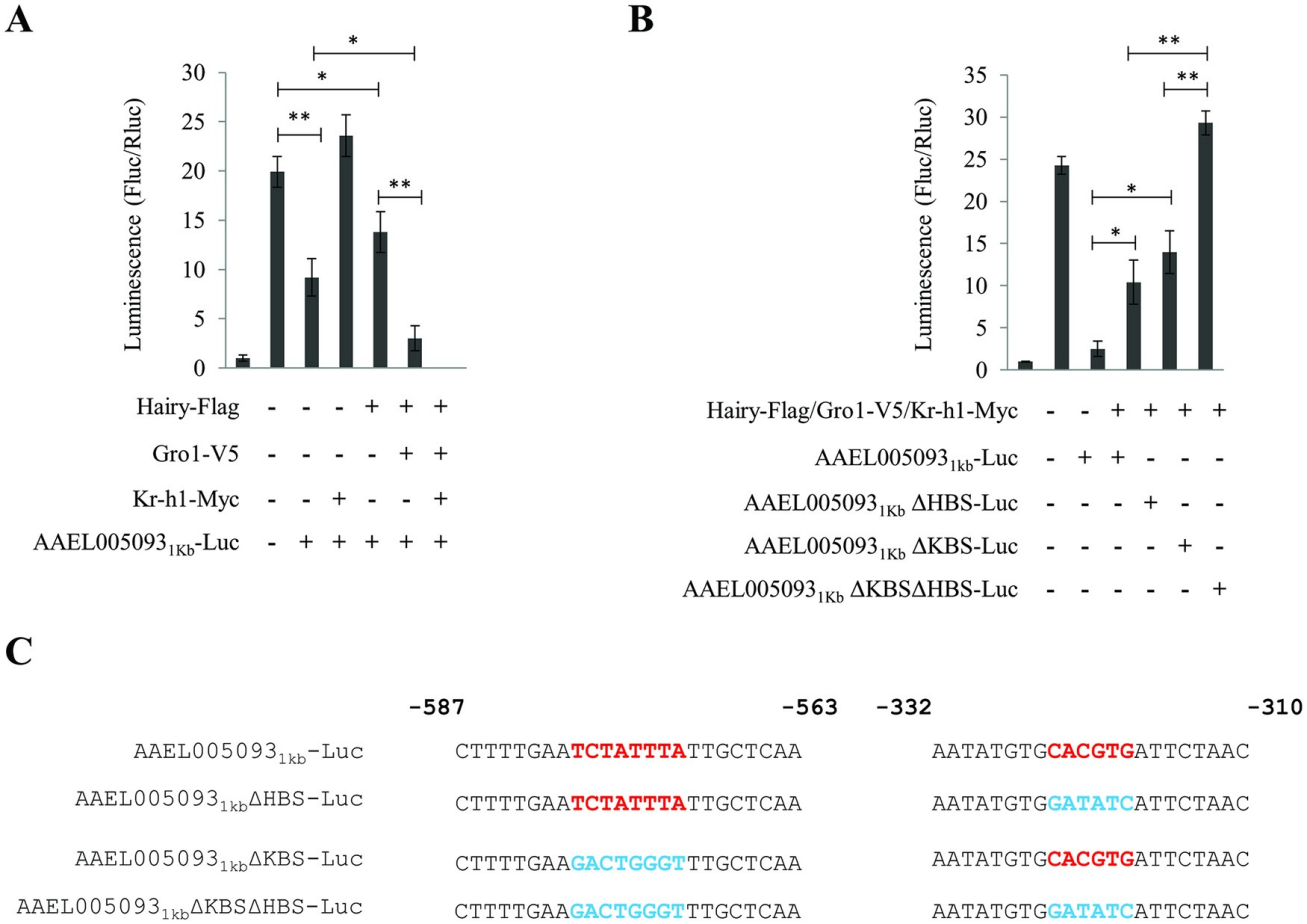
Synergistic action of Kr-h1 and Hairy in the repression of target genes downstream of JH/Met. **(A)** Luciferase reporter assays after co-transfection of expression vectors Hairy-Flag and/or Gro1-V5 and/or Kr-h1-Myc along with reporter construct AAEL005093_1kb_-Luc. Treatments with no input DNA and empty expression vector served as controls. Repression of promoter activity was observed when either Kr-h1-Myc or Hairy-Flag was overexpressed in the cell culture system. Co-repressor Gro1-V5 was required for the successful functioning of Hairy, as previously demonstrated in Saha et al. (2016) [22]. However, overexpression of both Kr-h1-Myc and Hairy-Flag (along with Gro1-V5) dramatically enhanced the intensity of repression of luciferase activity. Error bars represent ± SD. *p < 0.05; ***p < 0.001. **(B)** Mutation analysis of AAEL005093 promoter by luciferase reporter assays. The Kr-h1 and Hairy binding sites (KBS and HBS, respectively) in the AAEL005093_1kb_-Luc reporter construct were mutated either separately (AAEL005093_1kb_ ΔHBS-Luc and AAEL005093_1kb_ ΔKBS-Luc) or together (AAEL005093_1kb_ ΔKBSΔHBS-Luc) and co-transfected along with expression vectors Hairy-Flag and Gro1-V5 and Kr-h1-Myc. The repression in the luciferase activity observed with the AAEL005093_1kb_-Luc construct was partially compromised with the mutation of either KBS or HBS. A complete loss of repression in the promoter activity was observed when both KBS and HBS were mutated in the promoter upstream of the luciferase gene in reporter construct. Error bars represent ± SD. *p < 0.05; ***p < 0.001. **(C)** Predicted KBS and HBS along with their flanking regions harbored within 1kb of the AAEL005093 promoter and the various promoter mutations utilized in **(A)** and **(B)** are indicated.

In order to test the hypothesis about the synergistic action of Hairy and Kr-h1 in the repression of JH/Met target genes, we performed cell culture-based luciferase reporter assays. We used the regulatory region 1-kb 5´ upstream of the Met/Kr-h1/Hairy-repressed gene *AAEL005093* that harbors both KBSs and HBSs, within 255 bps of each other (Fig 5C). This upstream region was cloned into the luciferase reporter vector pGL3basic (AAEL005093_1kb_-Luc). The full-length *A*. *aegypti* Kr-h1 was amplified by PCR and cloned into pAc5.1 vector with a C-terminal Myc tag (Kr-h1-Myc). Previously cloned Hairy-Flag and Gro1-V5 [22] were also utilized in this study. Gro1-V5 was included because it has been demonstrated to be a necessary co-repressor for the successful functioning of Hairy in *A*. *aegypti* mosquitoes [22]. A 15- to 20-fold basal induction in the reporter activity was observed when AAEL005093_1kb_-Luc was transfected into *Drosophila melanogaster* S2 cells, rendering this experimental system feasible to address gene repression (Fig 6A). Co-transfection of either Kr-h1-Myc or Hairy-Flag and Gro1-V5 along with AAEL005093_1kb_-Luc resulted in significant repression in the promoter activity (Fig 6A). However, the level of repression was dramatically affected when 100 ng of Hairy-Flag, Gro1-V5 and Kr-h1-Myc was transfected together with AAEL005093_1kb_-Luc, reducing the luciferase signal to almost background levels (Fig 6A).

To further verify our finding about the synergistic action of the two factors, we mutated Kr-h1 (KBS-5´TCTATTTAG3´) and Hairy (HBS-5´CACGTG3´) binding motifs in the promoter of the AAEL005093 gene. The binding sites were mutated by inserting restriction sites in place of HBS (AAEL005093_1kb_ΔHBS-Luc) or KBS (AAEL005093_1kb_ΔKBS-Luc), or both (AAEL005093_1kb_ΔKBSΔHBS-Luc). Hairy-Flag, Gro1-V5 and Kr-h1-Myc were co-transfected with either AAEL005093_1kb_-Luc (as control) or its mutant versions (AAEL005093_1kb_ΔHBS-Luc, AAEL005093_1kb_ΔKBS-Luc and AAEL005093_1kb_ΔKBSΔHBS-Luc). In the control AAEL005093_1kb_-Luc transfection, a more than tenfold repression was observed in the presence of Hairy, Gro1 and Kr-h1 (Fig 6B). A partial loss of repression in the promoter activity was noted when either AAEL005093_1kb_ΔHBS-Luc or AAEL005093_1kb_ΔKBS-Luc reporter plasmids were co-transfected along with Hairy-Flag, Gro1-V5 and Kr-h1-Myc (Fig 6B). However, transfection of S2 cells with the reporter plasmid AAEL005093_1kb_ΔKBSΔHBS-Luc harboring mutations for both HBSs and KBSs in the presence of Hairy-Flag, Gro1-V5 and Kr-h1-Myc resulted in a complete loss of repression of the promoter activity (Fig 6B). The observed luciferase signal under the above experimental condition is comparable to the control samples transfected with the non-mutated reported plasmid AAEL005093_1kb_-Luc only, and lacked expressed Hairy, Gro1 or Kr-h1. The schematic representation of the reporter constructs with the HBSs and KBSs and the various mutations used for the luciferase transfection assays are provided in Fig 6C. The expression of tagged proteins Hairy-Flag, Gro1-V5 and Kr-h1-Myc in the cell culture system was confirmed by means of immunoblots using anti-Flag, anti-V5 and anti-Myc antibodies (S5A and S5B Fig). Using the same experimental approach, a synergistic action of Hairy and Kr-h1 on the promoter of the Met/Hairy/Kr-h1 target gene *AAEL006978* has also been demonstrated (S6A, S6B and S6C Fig), providing further support for our hypothesis.

### Hairy and Kr-h1 binds to specific sites in the promoter region of the target gene

**Fig 7:**
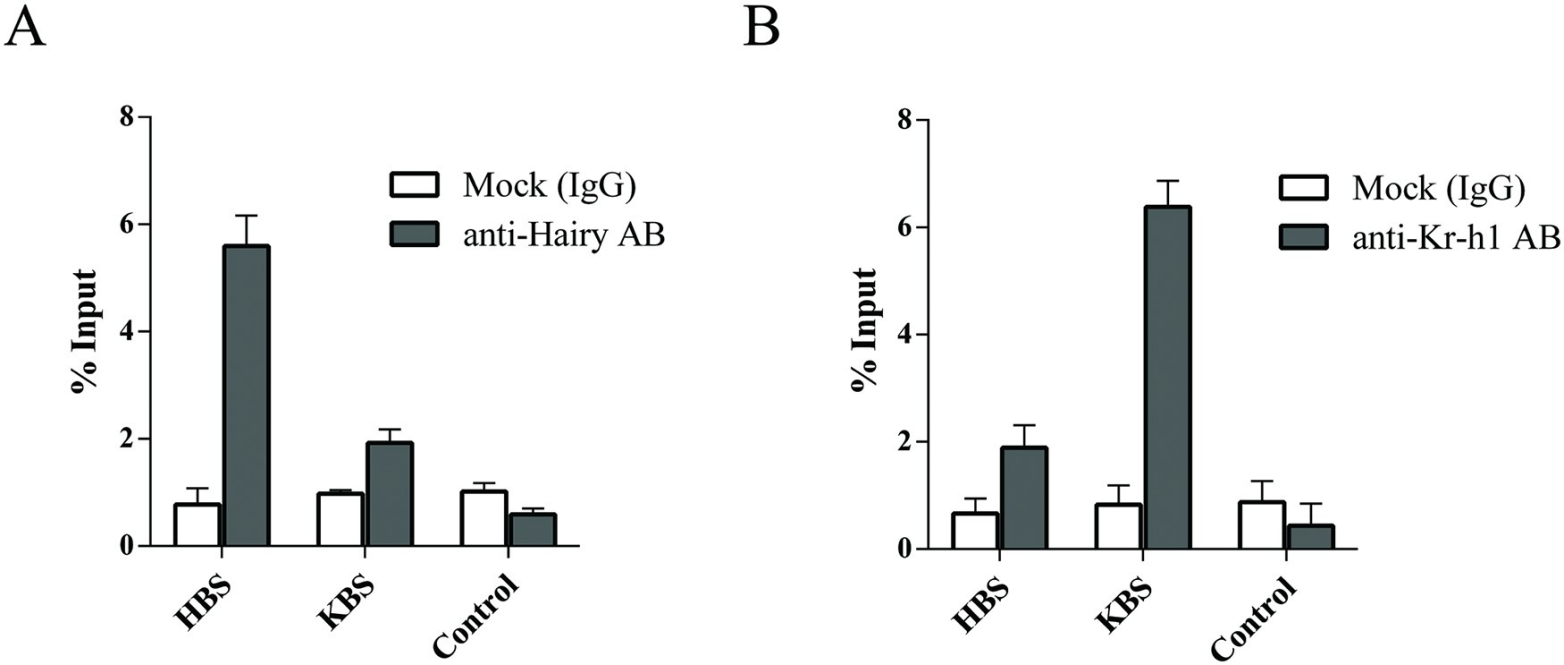
ChIP assays demonstrating the binding of Hairy and Kr-h1 proteins to specific regions in the promoter of target gene. **(A and B)** The promoter of Met/Hairy/Kr-h1 target gene *AAEL005093* was cloned (AAEL005093_1kb_-Luc) and transected into *Drosophila* S2 cells along with plasmids overexpressing both Hairy and Kr-h1 (Hairy-Flag and Kr-h1-Myc). ChIP were performed with *Aedes* anti-Hairy **(A)** and anti-Kr-h1 **(B)** antibody. anti-IgG antibody was used as mock control. Quantifications were performed by qRT-PCR using primer pairs targeting HBS and KBS regions in the target gene promoters. Primers targeting the plasmid backbone were utilized as controls. Data was represented as % of input DNA. Error bars represent ± SD.

To confirm the binding of the two factors Hairy and Kr-h1 to specific binding sites in the promoter of Met/Hairy/Kr-h1 target gene, we performed chromatin immunoprecipitation (ChIP) assays for the gene *AAEL005093*. The experiments were conducted in the cell culture system, using *Drosophila* S2 cells transected with AAEL005093_1kb_-Luc, Kr-h1-Myc and Hairy-Flag. ChIP assays were performed using *Aedes* anti-Hairy and anti-Kr-h1 antibody following a modified protocol specifically designed for S2 cells [33]. IP with an IgG antibody was used as control. Quantifications were performed by qRT-PCR using specific primers targeting the HBS and KBS locations in the cloned promoter of gene *AAEL005093*. An enrichment of HBS but not the KBS region was observed in ChIP assays using anti-Hairy antibody (Fig 7A). Similarly, use of anti-Kr-h1 antibody resulted in a substantial enrichment of the KBS site but not the HBS site (Fig 7B). These results clearly confirmed the physical binding of Hairy-HBS and Kr-h1-KBS sites in the target gene promoter. A primer targeting the plasmid backbone was utilized as control for both the experiments (Fig 7A and 7B).

### Effect of Hairy and Kr-h1 double knockdown on ovarian follicle growth

**Fig 8:**
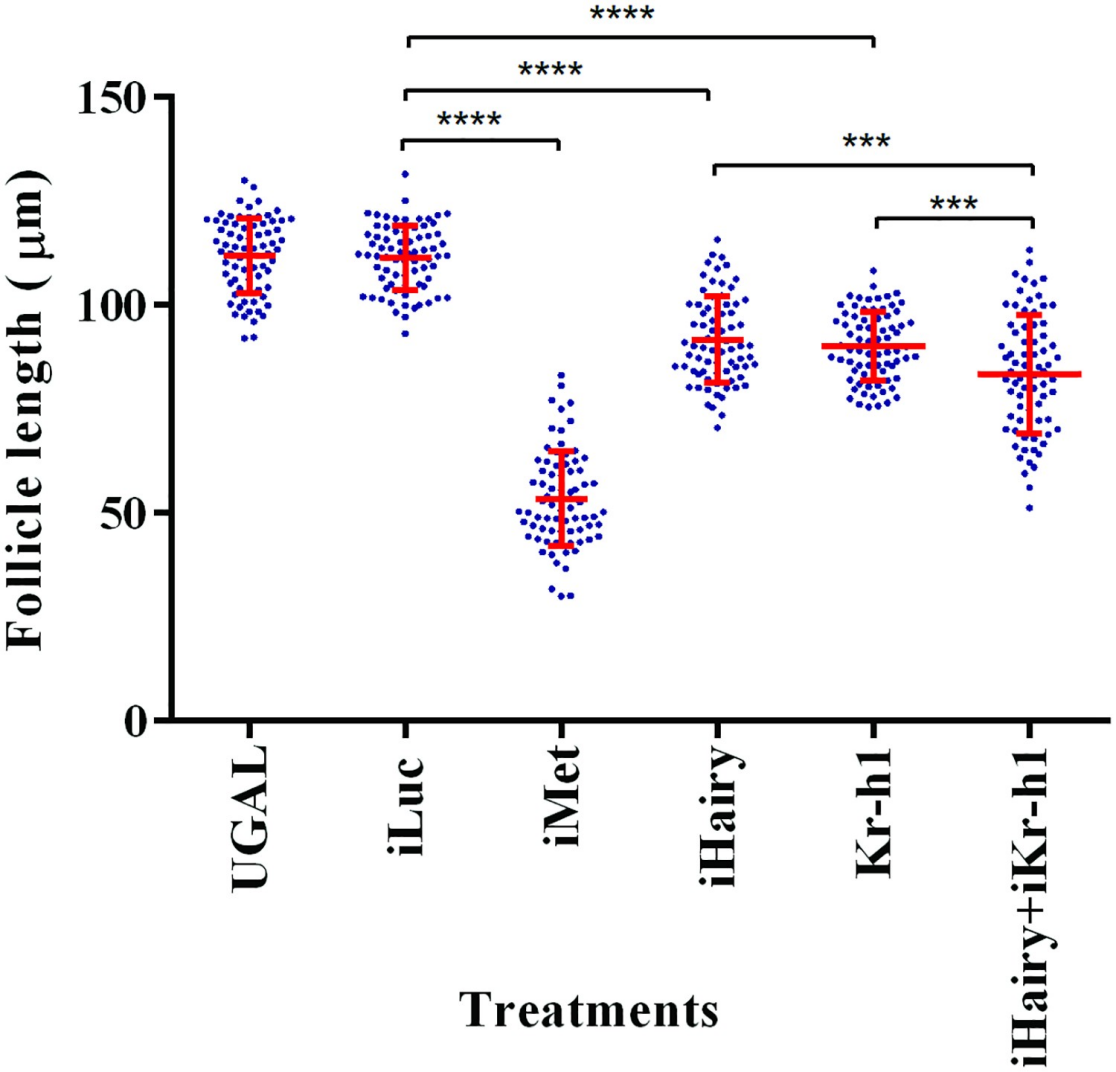
Effect of double knockdown of Hairy and Kr-h1 on the primary ovarian follicles. **(A)** Measurements of primary ovarian follicle length in *iMet*, *iHairy*, *iKr-h1* and *iHairy*+*iKr-h1* mosquitoes at 72h PE. *iLuc* and noninjected (UGAL) mosquitoes were used as controls. All measurements are in micrometers. Error bars represent ± SD. ***p < 0.001; ****p<0.0001.

Kr-h1 is a downstream factor of Met in the JH signaling pathway for gene repression. Thus, we checked the ovarian primary follicle length at 72h PE in *iKr-h1* mosquitoes. A reduction (approximately 20%) in the length of primary follicles was observed in *iKr-h1* mosquitoes when compared with iLuc, indicating the important role of Kr-h1 in PE follicle maturation (Fig 8). However, RNAi knockdown of *Met* had a more dramatic effect with an observed 52% reduction of follicle length (Fig 8). The *iKr-h1* had a phenotype very similar to that observed for *iHairy* mosquitoes, where a difference was observed between follicles of iHairy and *iMet* mosquitoes [22]. We then checked the follicle length of *iKr-h1*+*iHairy* double knockdown mosquitoes. A greater reduction in follicle length was observed in *iKr-h1*+*iHairy* samples than in single knockdowns of *iHairy* or *iKr-h1* samples, suggesting synergistic action of these factors (Fig 8). However, in iMet mosquitoes, a much greater reduction was observed. This could be due to the central role of Met in the JH signaling pathway. In addition to acting through Hairy and Kr-h1, Met regulates multiple genes directly [15, 17].

## Discussion

In female mosquitoes, JH is responsible for developmental events after adult eclosion during the PE phase. This JH-controlled phase is critical for a female mosquito to become competent for blood feeding and subsequent egg maturation. PE is characterized by sequential waves of gene expression, the most important of which are the early (EPE) and late (LPE) gene cohorts, exhibiting their respective peaks at 6–12 h and 72 h PE, which correlate with low and high JH titers, respectively [15, 24]. At its high titer, JH downregulates the expression of the EPE genes and upregulates the LPE genes. Zou et al. (2013) [15] have shown that the JH receptor Met mediates induction of the LPE genes via direct binding to their promoters, and later work confirmed the direct upregulation of selected LPE genes by Met [34]. Zou et al. (2013) also postulated that Met downregulates EPE genes indirectly requiring the involvement of intermediate TFs [15]. Indeed, our recent study demonstrated that the Met-regulated TF Hairy acts as a DNA-interacting transcriptional repressor that recruits a co-repressor Groucho and downregulates EPE gene expression [22]. Here, we have demonstrated the role of the zinc-finger domain protein Kr-h1 as a transcriptional repressor of a larger cohort of EPE genes downstream of Met. In a temporal microarray analysis, Kr-h1 has been classified as the LPE gene [15]. The molecular analysis utilizing *in-vivo* and *in-vitro* experiments has established Kr-h1 as an intermediate factor of the JH/Met gene-repression hierarchy. Furthermore, we were able to characterize a mosquito Kr-h1 binding site from our Kr-h1 repressed gene set, which was validated by EMSA analysis. It is worth mentioning that the same 8-mers (KBS10-KBS21) are overrepresented in both Met/Kr-h1- and Met/Hairy/Kr-h1-repressed gene sets. Also, the binding site we identified and subsequently validated matches 9 bp of the 30-bp KBS core, identified in the promoters of *B*. *mori* genes [30, 32]. Recently, using a chromatin immunoprecipitation (ChIP)-based approach, Ojani et al. (2018) identified a 12-bp-long DNA binding site for *A*. *aegypti* Kr-h1 [23]. The sequence shares similarities with the 30-mer KBS core from promoters of *B*. *mori* genes [30, 32] on which our bioinformatics analysis is based. The predicted sequence sits upstream of the 9-mer KBS that we identified and validated through the EMSA analysis. In their analysis, Ojani et al. (2018) did not rule out the possibility of the 9-mer sequence “TAAATAGA” working as a Kr-h1 binding site [23].

As a component of the JH signaling pathway, the role of Kr-h1 in reproduction has been investigated in several insects [35]. JH plays various roles in insect reproduction, and unlike mosquitoes where it guides a developmental phase (PE) in preparation for vitellogenesis, it induces vitellogenesis and oogenesis in other insects. In the orthopteran *Locusta migratoria*, JH-Met-Kr-h1 controls vitellogenesis and oocyte maturation in females [36]. In this insect, Kr-h1 is involved in regulation of vitellogenin gene expression in the fat body, lipid accumulation in primary oocyte, development of follicular epithelium, and ovarian growth [36]. However, in the hemipteran linden bug *Pyrrhocoris apterus* and the coleopteran beetle *T*. *castaneum*, no clear involvement of Kr-h1 in reproduction could be ascertained [37, 38]. Ojani et al. (2018) have implicated Kr-h1 in gene activation and repression in *A*. *aegypti* female mosquitoes [23]. Our data corroborates their observations by showing that Kr-h1 plays a crucial role in mosquito reproduction, functioning as an intermediate repressor downstream of Met in the JH signaling hierarchy during PE development. It remains to be elucidated whether Kr-h1 requires co-factors to accomplish a repressive or an activating role in regulation of gene expression.

We report hereby that the two Met downstream factors, Hairy and Kr-h1, act synergistically coordinating the repression of a subset of JH/Met-regulated genes. Several lines of evidence suggest this interaction. Gene transcripts were induced at different levels by the dsRNA-mediated depletion of *Met*, *Hairy* or *Kr-h1*. Simultaneous knockdown of *Kr-h1* and *Hairy* resulted in levels of upregulation of common target genes that are comparable to those for *iMet*. This led us to hypothesize for a possible synergistic action of these two factors in co-repression of a subset of JH/Met-regulated genes. Observation from bioinformatics analysis showed that within the promoters of about two-thirds of the Met/Hairy/Kr-h1-repressed genes, with both KBSs and HBSs, the two sites were very close to each other. The proximity of the two sites was not observed in the promoters of the Met/Hairy and the Met/Kr-h1 repressed gene sets, further strengthening our hypothesis of synergistic action. Cell culture-based luciferase reporter assays confirmed the synergistic action of these two factors. Furthermore, the necessity of these factors for the synergistic repression was confirmed when the complete loss of a target gene repression could only be observed with the mutation of both HBSs and KBSs in the regulatory region of the said gene. Taken together, our findings have revealed a novel aspect of the JH gene-repression hierarchy by demonstrating that Hairy and Kr-h1 synergistically repress a subset of JH/Met downregulated target genes. It appears that the synergistic action of Kh-h1 and Hairy is dictated by a target gene structure and requires the presence of corresponding binding sites for these two Met downstream TFs in regulatory regions of common target genes. It should be pointed out that Kr-h1 and Hairy together regulate about 20% (329 of 1613 total) of Met repressed genes (Fig 2A), indicating that there are additional factors involved in the JH/Met gene repression pathway, the identity of which remains unknown. This observation further confirms that gene regulation by JH is a complex phenomenon requiring a network of downstream factors.

Synergistic or cooperative regulation of gene expression is a widely occurring phenomenon [39–43]. In most cases, TFs responsible for synergistic or cooperative regulation of gene expression co-occupy regulatory regions of common target genes. Furthermore, synergistic TF action takes place in both gene activation [39, 41, 44, 45] and gene repression [43, 44, 46, 47]. In *Drosophila*, members of the ETS family of TFs have been shown to collaboratively repress the *even skipped* gene [47]. Interestingly, the co-repressor Groucho, which is a component of the Hairy/Kr-h1-mediated synergistic suppression of JH/Met target genes, is also required for *even skipped* repression [47]. Additionally, the Krüppel-like family of TFs has been implicated in the synergistic regulation of gene expression in conjunction with other TFs [45].

The TFs that are involved in synergistic or cooperative gene regulation are often observed interacting directly with each other [39, 48]. During *Drosophila* development, many TF-binding pairs are located in relatively short distances from each other on the DNA. *In-vitro* protein-protein binding experiments have shown that more than 65% of these TF pairs directly bind to each other, with some of them implicated in cooperative gene regulation [48]. Whether Kr-h1 and Hairy directly interact after their binding to a common target gene promoter remains to be determined.

## Material and methods

### Experimental animals

*A*. *aegypti* mosquito larvae were cultured at 27 °C in water supplemented with a mixture of yeast and rat chow (1:1 ratio). Adult mosquitoes were maintained in specifically designed cages at 27 °C, 86% humidity, and supplied with unlimited access to 10% (wt/vol) sucrose solution and water. All dissections were performed in *Aedes* physiological solution at room temperature [49]. Four-day-old adult females were blood fed on white Leghorn chicken. All procedures for the use of vertebrate animals were approved by the University of California, Riverside, Institutional Animal Care and Use Committee.

### dsRNA-mediated gene silencing

dsRNA-mediated knockdown of specific target genes was done following methods described by Zou et al. (2013) [15]. Briefly, dsRNA was synthesized using the MEGAscript kit (Ambion). The bacterial luciferase gene was used to generate control iLuc dsRNA. 0.6–0.8 μg of the desired dsRNA was injected into the thorax of cold-anesthetized female mosquitoes 1-day PE using the Picospritzer II (General Valve). For simultaneous knockdown of Hairy and Kr-h1, equal amounts (in μg) of both dsRNAs were mixed and the concentration of the dsHairy+dsKr-h1 was adjusted to 0.6–0.8 μg. Samples of the mosquito fat body (abdominal wall with adhered fat body) were collected 4 days post-injection. qRT-PCR was performed to verify the knockdown efficiency and specificity. All primers used are listed in S3 Table.

### Illumina RNA-seq library preparation, sequencing and bioinformatics analysis

The dsRNA-mediated knockdown, fat body sample collections and subsequent library preparation and sequencing for *iKr-h1* samples were performed as previously described [22]. RNAi for *luciferase* (*iLuc*) served as a control. Briefly, ten mosquito fat bodies were dissected at 4 days post-injection followed by RNA extraction using TRizol (Gibco/BRL). RNA-seq libraries were prepared using TruSeq RNA Library Preparation Kit (Illumina). Sequencing reactions were performed at the University of California Riverside Genomics Core Facility. Three independent biological replicates of the experiment were performed, resulting in three RNA-seq libraries per treatment. The obtained reads were aligned with Bowtie2 against *A*. *aegypti* transcript sequence database (AaegL1.3 geneset; Vectorbase). The resulting count table was transformed into values representing fragments per kilobase pair of transcripts per million fragments mapped (FPKM). Relative transcript abundance of iLuc and iKr-h1, defined as the sum of FPKM values of the two treatment samples, was sorted from highest to lowest. From the first set of RNA-seq experiments, 10,000 transcripts with the highest abundance were selected for further analysis. The differentially expressed transcripts were defined by a twofold increase or decrease of FPKM values.

### Ontology-based functional analysis

In order to examine the ontology of the genes, Evolutionary Genealogy of Genes: Non-supervised Orthologous Groups (eggNOG) database, version 3.0, was used, which is constructed through identification of reciprocal best BLAST matches and triangular linkage clustering [50]. The OGs are annotated with functional descriptions along with functional categories, which were derived from the original Clusters of Orthologous Groups/Eukaryotic Orthologous Groups categories. Adjustments of non-supervised orthologous groups (NOGs) were performed as described in Saha et al. (2016) [22].

### RNA extraction and qRT-PCR analysis

RNA was extracted from the fat bodies of six female mosquitoes using the TRIzol method (Invitrogen) according to the manufacturer’s protocol. It was concentrated using the RNeasy MiniElute cleanup kit (Qiagen) for further processing. RNA was treated with DNase I (Invitrogen), after which cDNAs were synthesized from 2 μg of this total RNA using the Omniscript Reverse Transcriptase kit (Qiagen). PCR was performed using the Platinum High Fidelity Supermix (Invitrogen). qRT-PCR was performed using the iCycler iQ system (Bio-Rad) and an IQ SYBR Green Supermix (Bio-Rad). Quantitative measurements were performed in triplicate and normalized to the internal control of actin mRNA for each sample. Real-time data were collected and exported to Excel (Microsoft) for analysis. RNA extraction, cDNA synthesis and subsequent qRT-PCR analysis were done as previously described [15]. All the primers used are listed in S3 Table.

### *In-vivo* JH treatment

Abdominal applications of JH to newly eclosed adult female mosquitoes (3 h PE) were performed as previously described [22]. Specifically, a 0.3-μL aliquot of 1 μg/mL JH III (Sigma) or solvent (acetone) was topically applied to the abdomen of newly emerged female mosquitoes (3 h PE). Sample collections were performed at 8h after hormonal treatment. The efficiency of hormonal treatment was tested by verifying the induction of the established JH-activated genes *Hairy* and *Kr-h1* using qRT-PCR.

### *In-vitro* fat body culture

Fat body tissue cultures with JH III and CHX supplementations were performed as previously described [22]. For RNAi-fat body tissue culture tandem experiments, dsRNA-mediated gene silencing was performed as described above, followed by fat body collections at 4 days post-injection. The dissected fat bodies were used for *in*-*vitro* fat body culture, with and without JH.

### Western blotting

cDNA encoding full-length *A*. *aegypti* Kr-h1 (AAEL002390) was cloned into pcDNA3.1 (Thermo Fisher, USA) and was sent out for antibody production. Sub-cloning, bacterial expression and the subsequent antibody production in mouse were performed by Genscript (USA). Antiserum against Kr-h1 was purified by means of affinity chromatography using the ImmunoPure IgG purification kit (Pierce). In addition to Kr-h1, previously generated antiserum against *A*. *aegypti* Hairy and Met were utilized in this study [22]. Fat-body protein sample collections and western blots were performed as previously described [51]. A specific mouse monoclonal antibody against β-actin (Sigma, USA) was used as loading control.

### Electrophoretic mobility shift assay (EMSA)

The DNA oligonucleotides containing the core motif and flanking region were annealed, purified and end-labeled with [γ-^32^P] ATP, as described previously [15]. Nuclear extracts from the mosquito fat body 48h PE were prepared using NE-PER Nuclear and Cytoplasmic Extraction Reagents (Thermo Scientific, Chino, CA) and were subsequently utilized for the EMSAs. Binding reactions were performed using the Gel Shift Assay System (Promega) and the DNA-protein complex was resolved on 5% TBE Criterion Precast Gel (Bio-Rad). Following electrophoresis, the gel was dried, exposed to phosphor imaging screens and visualized by means of autoradiography using the Personal Molecular Imager (Bio-Rad). For competition assays, 50-fold unlabeled specific motif or unlabeled SP1 (non-specific competitor oligonucleotides; Promega) motif was incubated with nuclear extract for 10 min and then further incubated with labeled motif for 20 min. Generated polyclonal *A*. *aegypti* Kr-h1 antibodies were used to test the presence of the TF in the observed DNA-protein complex.

### Luciferase reporter assays

*A*. *aegypti* Kr-h1 with a C-terminal Myc-tag was sub-cloned into the pAc5.1 vector (Kr-h1-Myc) (Thermo Scientific, Chino CA). Previously cloned *A*. *aegypti* Hairy-Flag and Gro1-V5 were also used in this experiment [22]. A 1-kb promoter regions of the target genes *AAEL005093* and *AAEL006978*, both harboring KBSs and HBSs, was PCR amplified and cloned into reporter vector PGL3basic (Promega), followed by sequence confirmation (AAEL005093_1kb_-Luc and AAEL006978_1kb_-Luc). Kr-h1 or Hairy binding motifs in both the cloned promoters were mutated by incorporating restriction sites in place of the defined KBS (AAEL005093_1kb_ΔKBS-Luc and AAEL006978_1kb_ ΔKBS-Luc) or HBS (AAEL005093_1kb_ΔHBS-Luc and AAEL006978_1kb_ ΔHBS-Luc), respectively. Mutation of both KBSs and HBSs was achieved by sequential incorporation of restriction sites replacing the binding motifs (AAEL005093_1kb_ΔKBSΔHBS-Luc and AAEL006978_1kb_ ΔKBSΔHBS-Luc). Transient transfection of cultured *Drosophila* S2 cells was performed using the FuGENE HD reagent (Promega) following manufacturer’s instructions. 100ng of desired reporter plasmids and 10ng of the control *Renilla* luciferase reporter vector *pCopia* were co-transfected into the S2 cells along with 200ng each of the expression plasmids Kr-h1-Myc, Hairy-Flag and Gro1-V5, in various combinations. The total concentration of transfected plasmid in each well was normalized by adding the empty expression vector pAc5.1. Synthesized dsRNA samples for *Dm*Kr-h1, *Dm*Hairy and *Dm*Groucho were supplemented in the transfection mixture as required, in order to negate the effects of endogenous factors on our experiment. Luciferase assays were performed using the Dual Luciferase Assay Kit (Promega), as previously described [22].

### Chromatin immunoprecipitation (ChIP) assay

ChIP assays were performed in *Drosophila* S2 cells as previously described [33]. Briefly, 5–10×10^6^ transfected S2 cells were fixed with 1% formaldehyde in tissue culture media for 10 min at room temperature followed by addition of glycine (0.125M) in order to stop the reaction. After washing in ice cold PBS (1X) for 2 mins, cells were re-suspended in cell lysis buffer (5 mM pH 8.0 PIPES buffer, 85 mM KCl, 0.5% Nonidet P40, and protease inhibitors cocktail) followed by the addition of nucleus lysis buffer (50 mM pH 8.1 Tris-HCl, 10 mM EDTA, 1% SDS and protease inhibitors cocktail). After a 20 min incubation at 4°C, the nuclear extract was sheared by sonication on ice for 5 min (pulsed 10 times for 30 s with 30 s intervals) using Athena Ultrasonic Processor (Probe Sonicator) ATP-150; Athena Technology Inc. The chromatin solution was then clarified by centrifugation at 14,000 rpm for 10 mins at 4°C. For immunoprecipitation 2μl of each *Aedes* anti-Hairy and anti-Krh1 were incubated with the chromatin for 2h followed by overnight binding to 50μl pre-washed protein A agarose beads at 4°C with shaking. Anti-IgG antibody was used as mock control. The beads were washed three times with wash buffer 1 (0.1% SDS, 1% Triton, 2 mM EDTA, 20 mM pH 8.0 Tris, and 150 mM NaCl); three times with wash buffer 2 (0.1% SDS, 1% Trition, 2 mM EDTA, 20 mM pH 8.0 Tris, and 500 mM NaCl); and two times with wash buffer 3 (10 mM pH 8.1 Tris, 1 mM EDTA, 0.25 M LiCl, 1% NP40, and 1% sodium deoxycholate). The immunoprecipitated DNA was eluted from the beads in 0.1 M NaHCO_3_ and 1% SDS followed by revrese cross-linking by incubating overnight at 65°C. DNA was extracted by phenol-chloroform method using ethanol precipitation. Quantifications were performed by qRT-PCR and represented as % of input DNA. A primer pair targeting the plasmid backbone was used as control. The primers used are mentioned in the S3 Table.

## Supporting information

S1 Fig Reproducibility of the RNA-seq experiments. Venn-diagram showing the number of transcripts co-activated by the dsRNA-mediated depletion of *Kr-h1* (*iKr-h1*) in three different biological replicates, as identified by RNA-seq analysis of the female mosquito fat body. With 207 common transcripts, there was a high degree of overlap among the three replicates, indicating reproducibility of the parallel experiments.
(TIF)

S2 Fig qRT-PCR based analysis of the expression of Met/Hairy/Kr-h1 target gene *AAEL006978*. **(A)** Hormonal application experiments showing the effect of JH on the expression of *AAEL006978 (protein-glutamine gamma-glutamyltransferase)*. A clear repression of the tested gene was observed in JH-treated samples. NT, no treatment. **(B)** The necessity of intermediate factors for the JH-mediated repression of the target gene *AAEL006978* as determined by *in-vitro* fat body culture experiments. JH-mediated repression was compromised by the addition of CHX into the tissue culture medium (JH+CHX). Solvent (acetone) and CHX-treated samples were used as controls. **(C)** qRT-PCR-based expression analysis of Met/Hairy/Kr-h1 target gene *AAEL006978* in *iMet*, *iHairy*, *iKr-h1* and *iHairy*+*iKr-h1* samples. iLuc samples were used as controls. Error bars represent ± SD. *p < 0.05; ***p < 0.001.
(TIF)

S3 Fig Ontology analysis of the Met/Hairy/Kr-h1 repressed gene cohort. All 130 transcripts activated in the fat body by dsRNA-mediated knockdown of *Met*, *Hairy*, and *Kr-h1* were mapped to get an overview of the functional groups affected by the transcription factors. Two major categories Cellular Processes and Signaling (*left*) and Metabolism (*right*) are shown as pie diagrams. Functional groups with corresponding abbreviations and colors are indicated. The other major category- Information Storage and Processing (not shown in the graph) was not represented in the iMet/iHairy/iKr-h1-activated gene set, with no transcript mapped to the subcategories under this heading.
(TIF)

S4 Fig Frequency of potential *Aa*Kr-h1 binding site in the promoter sequences of Met/Kr-h1/Hairy repressed genes. The graph showing the frequency of hits for all the 8-mer sequences generated for the characterization of potential Kr-h1 binding site (KBS) (from Fig 5A) in the region 5-kb upstream of *iMet*/*iKr-h1*/*iHairy* upregulated transcripts. The 23 8-mers used are listed in Fig 5A.
(TIF)

S5 Fig Expression analysis of tagged proteins in the cell culture system. **(A and B)** Western blots showing the expression of tagged proteins Kr-h1-Myc, Hairy-Flag and Gro1-V5 in cell culture samples in Fig 6A and 6B, respectively. Commercially available anti-Myc, Flag and V5 antibodies were utilized for the Western blot analysis. Actin was used as a loading control.
(TIF)

S6 Fig Cell culture-based luciferase reporter assay of cloned 1kb promoter region of the gene *AAEL006978*. **(A)** Luciferase reporter assays after co-transfection of expression vectors Hairy-Flag and/or Gro1-V5 and/or Kr-h1-Myc along with reporter construct AAEL006978_1kb_-Luc. Treatments with no input DNA and empty expression vector served as controls. Repression of promoter activity was observed when either Kr-h1-Myc or Hairy-Flag was overexpressed in the cell culture system. Co-repressor Gro1-V5 was required for the successful functioning of Hairy. However, overexpression of both Kr-h1-Myc and Hairy-Flag (along with Gro1-V5) dramatically enhanced the intensity of repression of luciferase activity. Error bars represent ± SD. *p < 0.05; ***p < 0.001. **(B)** Mutation analysis of AAEL006978 promoter by luciferase reporter assays. The Kr-h1 and Hairy binding sites (KBS and HBS, respectively) in the AAEL006978_1kb_-Luc reporter construct were mutated either separately (AAEL006978_1kb_ ΔHBS-Luc and AAEL006978_1kb_ ΔKBS-Luc) or together (AAEL006978_1kb_ ΔKBSΔHBS-Luc) and co-transfected along with expression vectors Hairy-Flag and Gro1-V5 and Kr-h1-Myc. The repression in the luciferase activity observed with the AAEL006978_1kb_-Luc construct was partially compromised with the mutation of either KBS or HBS. A complete loss of repression in the promoter activity was observed when both KBS and HBS were mutated in the promoter upstream of the luciferase gene in reporter construct. Error bars represent ± SD. *p < 0.05; ***p < 0.001. **(C)** Predicted KBS and HBS along with their flanking regions harbored within 1kb of the AAEL006978 promoter and the various promoter mutations utilized in **(A)** and **(B)** are indicated.
(TIF)

S1 Dataset List of transcripts upregulated by the dsRNA mediated knockdown of *Kr-h1* in the fat body of adult female mosquito, *A*. *aegypti*. The list of transcripts upregulated in *iKr-h1* and in *iMet* mosquito fat body (overlapped transcripts between *iKr-h1*↑ and *iMet*↑) are provided as well. The dataset includes the list of 10,000 transcripts with the highest abundance, utilized for downstream analysis of *iKr-h1* upregulated transcripts.
(XLSX)

S2 Dataset RNA-seq transcriptomic data for the two other biological replicates. List of 10,000 most abundant transcripts, *iKr-h1* upregulated transcripts and the overlapped transcripts between *iKr-h1*↑ and *iMet*↑ for the biological replicate 2 and 3 are provided.
(XLSX)

S3 Dataset List of transcripts activated by the dsRNA mediated depletion of Kr-h1 (*iKr-h1*) and Hairy (*iHairy*) in *iMet* background. The list shows the three way overlap between upregulated transcripts in three different RNA-seq libraries prepared from *iMet*, *iHairy* and *iKr-h1* adult female mosquito fat body.
(XLSX)

S1 Table Gene ontology based functional annotation of *iMet*/*iHairy*/*iKr-h1* activated genes. (XLSX)

S2 Table Distance analysis between Kr-h1 and Hairy binding sites in the promoters of target genes repressed by both the transcription factors. (XLSX)

S3 Table List of primer sequences utilized in this study. (XLSX)

## References

[1] Jindra M, Palli SR, Riddiford LM (2013) The juvenile hormone signaling pathway in insect development. Annu Rev Entomol 58: 181–204. 10.1146/annurev-ento-120811-153700 22994547

[2] Jindra M, Bellés X, Shinoda T (2015) Molecular basis of juvenile hormone signaling. Curr Opin Insect Sci 11: 39–46. 10.1016/j.cois.2015.08.004 28285758

[3] Wilson TG, Fabian J (1986) A Drosophila melanogaster mutant resistant to a chemical analog of juvenile hormone. Dev Biol 118: 190–201. 10.1016/0012-1606(86)90087-4 3095161

[4] Ashok M, Turner C, Wilson TG (1998) Insect juvenile hormone resistance gene homology with the bHLH-PAS family of transcriptional regulators. Proc Natl Acad Sci USA 95: 2761–2766. 10.1073/pnas.95.6.2761 9501163 PMC19642

[5] Miura K, Oda M, Makita S, Chinzei Y (2005) Characterization of the Drosophila Methoprene -tolerant gene product. Juvenile hormone binding and ligand-dependent gene regulation. FEBS J 272: 1169–1178. 10.1111/j.1742-4658.2005.04552.x 15720391

[6] Konopova B, Jindra M (2007) Juvenile hormone resistance gene Methoprene-tolerant controls entry into metamorphosis in the beetle Tribolium castaneum. Proc Natl Acad Sci USA 104: 10488–10493. 10.1073/pnas.0703719104 17537916 PMC1965540

[7] Charles JP, Iwema T, Epa VC, Takaki K, Rynes J, et al (2011) Ligand-binding properties of a juvenile hormone receptor, Methoprene-tolerant. Proc Natl Acad Sci USA 108: 21128–21133. 10.1073/pnas.1116123109 22167806 PMC3248530

[8] Jindra M, Uhlirova M, Charles JP, Smykal V, Hill RJ (2015) Genetic Evidence for Function of the bHLH-PAS Protein Gce/Met As a Juvenile Hormone Receptor. PLoS Genet 11: e1005394 10.1371/journal.pgen.1005394 26161662 PMC4498814

[9] Li M, Mead EA, Zhu J (2011) Heterodimer of two bHLH-PAS proteins mediates juvenile hormone-induced gene expression. Proc Natl Acad Sci USA 108: 638–643. 10.1073/pnas.1013914108 21187375 PMC3021087

[10] Zhang Z, Xu J, Sheng Z, Sui Y, Palli SR (2011) Steroid receptor co-activator is required for juvenile hormone signal transduction through a bHLH-PAS transcription factor, methoprene tolerant. J Biol Chem 286: 8437–8447. 10.1074/jbc.M110.191684 21190938 PMC3048728

[11] Li M, Liu P, Wiley JD, Ojani R, Bevan DR, et al (2014) A steroid receptor coactivator acts as the DNA-binding partner of the methoprene-tolerant protein in regulating juvenile hormone response genes. Mol Cell Endocrinol 394: 47–58. 10.1016/j.mce.2014.06.021 25004255 PMC4163509

[12] Kayukawa T, Minakuchi C, Namiki T, Togawa T, Yoshiyama M, et al (2012) Transcriptional regulation of juvenile hormone-mediated induction of Krüppel homolog 1, a repressor of insect metamorphosis. Proc Natl Acad Sci USA 109: 11729–11734. 10.1073/pnas.1204951109 22753472 PMC3406821

[13] Shin SW, Zou Z, Saha TT, Raikhel AS (2012) bHLH-PAS heterodimer of methoprene-tolerant and Cycle mediates circadian expression of juvenile hormone-induced mosquito genes. Proc Natl Acad Sci USA 109: 16576–16581. 10.1073/pnas.1214209109 23012454 PMC3478602

[14] Kayukawa T, Tateishi K, Shinoda T (2013) Establishment of a versatile cell line for juvenile hormone signaling analysis in Tribolium castaneum. Sci Rep 3: 1570 10.1038/srep01570 23535851 PMC3610134

[15] Zou Z, Saha TT, Roy S, Shin SW, Backman TW, et al (2013) Juvenile hormone and its receptor, methoprene-tolerant, control the dynamics of mosquito gene expression. Proc Natl Acad Sci USA 110: E2173–2181. 10.1073/pnas.1305293110 23633570 PMC3683779

[16] Cui Y, Sui Y, Xu J, Zhu F, Palli SR (2014) Juvenile hormone regulates Aedes aegypti Kruppel homolog 1 through a conserved E box motif. Insect Biochem Mol Biol 52: 23–32. 10.1016/j.ibmb.2014.05.009 24931431 PMC4143451

[17] Wang JL, Saha TT, Zhang Y, Zhang C, Raikhel AS (2017) Juvenile hormone and its receptor methoprene-tolerant promote ribosomal biogenesis and vitellogenesis in the Aedes aegypti mosquito. J Biol Chem 292: 10306–10315. 10.1074/jbc.M116.761387 28446607 PMC5473233

[18] He Q, Wen D, Jia Q, Cui C, Wang J, et al (2014) Heat shock protein 83 (Hsp83) facilitates methoprene-tolerant (Met) nuclear import to modulate juvenile hormone signaling. J Biol Chem 289: 27874–27885. 10.1074/jbc.M114.582825 25122763 PMC4183821

[19] He Q, Zhang Y, Zhang X, Xu D, Dong W, et al (2017) Nucleoporin Nup358 facilitates nuclear import of Methoprene-tolerant (Met) in an importin beta- and Hsp83-dependent manner. Insect Biochem Mol Biol 81: 10–18. 10.1016/j.ibmb.2016.12.005 27979731

[20] Liu P, Peng HJ, Zhu J (2015) Juvenile hormone-activated phospholipase C pathway enhances transcriptional activation by the methoprene-tolerant protein. Proc Natl Acad Sci USA 112: E1871–1879. 10.1073/pnas.1423204112 25825754 PMC4403202

[21] Ojani R, Liu P, Fu X, Zhu J (2016) Protein kinase C modulates transcriptional activation by the juvenile hormone receptor methoprene-tolerant. Insect Biochem Mol Biol 70: 44–52. 10.1016/j.ibmb.2015.12.001 26689644 PMC4767628

[22] Saha TT, Shin SW, Dou W, Roy S, Zhao B, et al (2016) Hairy and Groucho mediate the action of juvenile hormone receptor Methoprene-tolerant in gene repression. Proc Natl Acad Sci USA 113: E735–743. 10.1073/pnas.1523838113 26744312 PMC4760797

[23] Ojani R, Fu X, Ahmed T, Liu P, Zhu J (2018) Krüppel homologue 1 acts as a repressor and an activator in the transcriptional response to juvenile hormone in adult mosquitoes. Insect Mol Biol 27: 268–278. 10.1111/imb.12370 29314423 PMC5837916

[24] Zhao B, Hou Y, Wang J, Kokoza VA, Saha TT, et al (2016) Determination of juvenile hormone titers by means of LC-MS/MS/MS and a juvenile hormone-responsive Gal4/UAS system in *Aedes aegypti* mosquitoes. Insect Biochem Mol Biol 77: 69–77. 10.1016/j.ibmb.2016.08.003 27530057 PMC5028310

[25] Pecasse F, Beck Y, Ruiz C, Richards G (2000) Krüppel-homolog, a stage-specific modulator of the prepupal ecdysone response, is essential for Drosophila metamorphosis. Dev Biol 221: 53–67. 10.1006/dbio.2000.9687 10772791

[26] Minakuchi C, Zhou X, Riddiford LM (2008) Krüppel homolog 1 (Kr-h1) mediates juvenile hormone action during metamorphosis of Drosophila melanogaster. Mech Dev 125: 91–105. 10.1016/j.mod.2007.10.002 18036785 PMC2276646

[27] Minakuchi C, Namiki T, Shinoda T (2009) Krüppel homolog 1, an early juvenile hormone-response gene downstream of Methoprene-tolerant, mediates its anti-metamorphic action in the red flour beetle Tribolium castaneum. Dev Biol 325: 341–350. 10.1016/j.ydbio.2008.10.016 19013451

[28] Bellés X, Santos CG (2014) The MEKRE93 (Methoprene tolerant-Krüppel homolog 1-E93) pathway in the regulation of insect metamorphosis, and the homology of the pupal stage. Insect Biochem Mol Biol 52: 60–68. 10.1016/j.ibmb.2014.06.009 25008785

[29] Ureña E, Manjón C, Franch-Marro X, Martín D (2014) Transcription factor E93 specifies adult metamorphosis in hemimetabolous and holometabolous insects. Proc Natl Acad Sci USA 111: 7024–7029. 10.1073/pnas.1401478111 24778249 PMC4024875

[30] Kayukawa T, Nagamine K, Ito Y, Nishita Y, Ishikawa Y, et al (2016) Krüppel homolog 1 inhibits insect metamorphosis via direct transcriptional repression of Broad-Complex, a pupal specifier gene. J Biol Chem 291: 1751–1762. 10.1074/jbc.M115.686121 26518872 PMC4722455

[31] Ureña E, Chafino S, Manjón C, Franch-Marro X, Martín D (2016) The occurrence of the holometabolous pupal stage requires the interaction between E93, Krüppel-Homolog 1 and Broad-Complex. PLoS Genet 12: e1006020 10.1371/journal.pgen.1006020 27135810 PMC4852927

[32] Kayukawa T, Jouraku A, Ito Y, Shinoda T (2017) Molecular mechanism underlying juvenile hormone-mediated repression of precocious larval–adult metamorphosis. Proc Natl Acad Sci USA 114: 1057–1062. 10.1073/pnas.1615423114 28096379 PMC5293048

[33] Zhang Y, Malone JH, Powell SK, Periwal V, Spana E, et al (2010) Expression in aneuploid Drosophila S2 cells. PLoS Biol 8: e1000320 10.1371/journal.pbio.1000320 20186269 PMC2826376

[34] Wang X, Hou Y, Saha TT, Pei G, Raikhel AS, et al (2017) Hormone and receptor interplay in the regulation of mosquito lipid metabolism. Proc Natl Acad Sci USA 114: E2709–E2718. 10.1073/pnas.1619326114 28292900 PMC5380040

[35] Roy S, Saha TT, Zou Z, Raikhel AS (2018) Regulatory pathways controlling female insect reproduction. Annu Rev Entomol 63: 489–511. 10.1146/annurev-ento-020117-043258 29058980

[36] Song J, Wu Z, Wang Z, Deng S, Zhou S (2014) Krüppel-homolog 1 mediates juvenile hormone action to promote vitellogenesis and oocyte maturation in the migratory locust. Insect Biochem Mol Biol 52: 94–101. 10.1016/j.ibmb.2014.07.001 25017142

[37] Parthasarathy R, Sheng Z, Sun Z, Palli SR (2010) Ecdysteroid regulation of ovarian growth and oocyte maturation in the red flour beetle, Tribolium castaneum. Insect Biochem Mol Biol 40: 429–439. 10.1016/j.ibmb.2010.04.002 20385235 PMC2916939

[38] Smykal V, Bajgar A, Provaznik J, Fexova S, Buricova M, et al (2014) Juvenile hormone signaling during reproduction and development of the linden bug, Pyrrhocoris apterus. Insect Biochem Mol Biol 45: 69–76. 10.1016/j.ibmb.2013.12.003 24361539

[39] Kawata Y, Suzuki H, Higaki Y, Denisenko O, Schullery D, et al (2002) bcn-1 Element-dependent activation of the laminin gamma 1 chain gene by the cooperative action of transcription factor E3 (TFE3) and Smad proteins. J Biol Chem 277: 11375–11384. 10.1074/jbc.M111284200 11801598

[40] Morin S, Pozzulo G, Robitaille L, Cross J, Nemer M (2005) MEF2-dependent recruitment of the HAND1 transcription factor results in synergistic activation of target promoters. J Biol Chem 280: 32272–32278. 10.1074/jbc.M507640200 16043483

[41] Nakayama K (2013) cAMP-response element-binding protein (CREB) and NF-κB transcription factors are activated during prolonged hypoxia and cooperatively regulate the induction of matrix metalloproteinase MMP1. J Biol Chem 288: 22584–22595. 10.1074/jbc.M112.421636 23775082 PMC3829345

[42] Perez-Pinera P, Ousterout DG, Brunger JM, Farin AM, Glass KA, et al (2013) Synergistic and tunable human gene activation by combinations of synthetic transcription factors. Nat Methods 10: 239–242. 10.1038/nmeth.2361 23377379 PMC3719416

[43] Delesque-Touchard N, Park SH, Waxman DJ (2000) Synergistic action of hepatocyte nuclear factors 3 and 6 on CYP2C12 gene expression and suppression by growth hormone-activated STAT5b. Proposed model for female specific expression of CYP2C12 in adult rat liver. J Biol Chem 275: 34173–34182. 10.1074/jbc.M004027200 10931833

[44] He X, Samee MA, Blatti C, Sinha S (2010) Thermodynamics-based models of transcriptional regulation by enhancers: the roles of synergistic activation, cooperative binding and short-range repression. PloS Comput Biol 6: e1000935 10.1371/journal.pcbi.1000935 20862354 PMC2940721

[45] Shao J, Yang VW, Sheng H (2008) Prostaglandin E2 and Krüppel-like transcription factors synergistically induce the expression of decay-accelerating factor in intestinal epithelial cells. Immunology 125: 397–407. 10.1111/j.1365-2567.2008.02847.x 18435741 PMC2669143

[46] Banerjee N, Zhang MQ (2003) Identifying cooperativity among transcription factors controlling the cell cycle in yeast. Nucleic Acids Res 31: 7024–7031. 10.1093/nar/gkg894 14627835 PMC290262

[47] Webber JL, Zhang J, Massey A, Sanchez-Luege N, Rebay I (2018) Collaborative repressive action of the antagonistic ETS transcription factors Pointed and Yan fine-tunes gene expression to confer robustness in Drosophila. Development 145: 165985. 10.1242/dev.165985 PMC6053666 29848501

[48] Kazemian M, Pham H, Wolfe SA, Brodsky MH, Sinha S (2013) Widespread evidence of cooperative DNA binding by transcription factors in Drosophila development. Nucleic Acids Res 41: 8237–8252. 10.1093/nar/gkt598 23847101 PMC3783179

[49] Roy SG, Hansen IA, Raikhel AS (2007) Effect of insulin and 20-hydroxyecdysone in the fat body of the yellow fever mosquito, Aedes aegypti. Insect Biochem Mol Biol 37: 1317–1326. 10.1016/j.ibmb.2007.08.004 17967350 PMC2104489

[50] Powell S, Szklarczyk D, Trachana K, Roth A, Kuhn M, et al (2012) eggNOG v3.0: orthologous groups covering 1133 organisms at 41 different taxonomic ranges. Nucleic Acids Res 40: D284–D289. 10.1093/nar/gkr1060 22096231 PMC3245133

[51] Hansen IA, Attardo GM, Roy SG, Raikhel AS (2005) Target of rapamycin-dependent activation of S6 kinase is a central step in the transduction of nutritional signals during egg development in a mosquito. J Biol Chem 280: 20565–20572. 10.1074/jbc.M500712200 15788394

