# Supplementary figures and images for: Synergistic action of the transcription factors Krüppel homolog 1 and Hairy in juvenile hormone/Methoprene-tolerant-mediated gene-repression in the mosquito Aedes aegypti
Source: PLoS Genet. 2019 Oct 29;15(10):e1008443. doi: 10.1371/journal.pgen.1008443 (PMC6818763; doi:10.1371/journal.pgen.1008443)

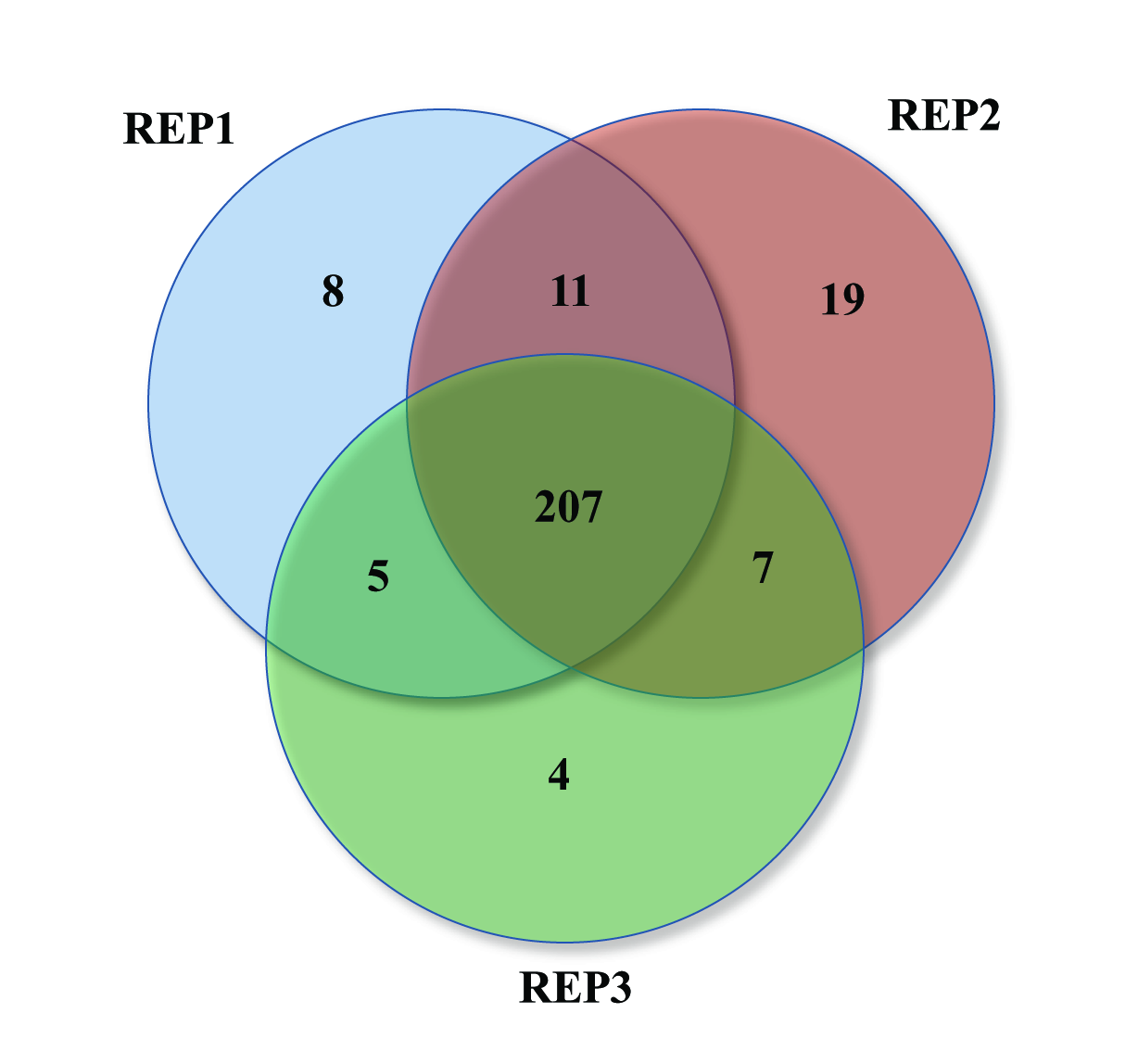

Supplement: S1 Fig — Venn-diagram showing the number of transcripts co-activated by the dsRNA-mediated depletion of Kr-h1 (iKr-h1) in three different biological replicates, as identified by RNA-seq analysis of the female mosquito fat body. With 207 common transcripts, there was a high degree of overlap among the three replicates, indicating reproducibility of the parallel experiments. (TIF) [file pgen.1008443.s001.tif]

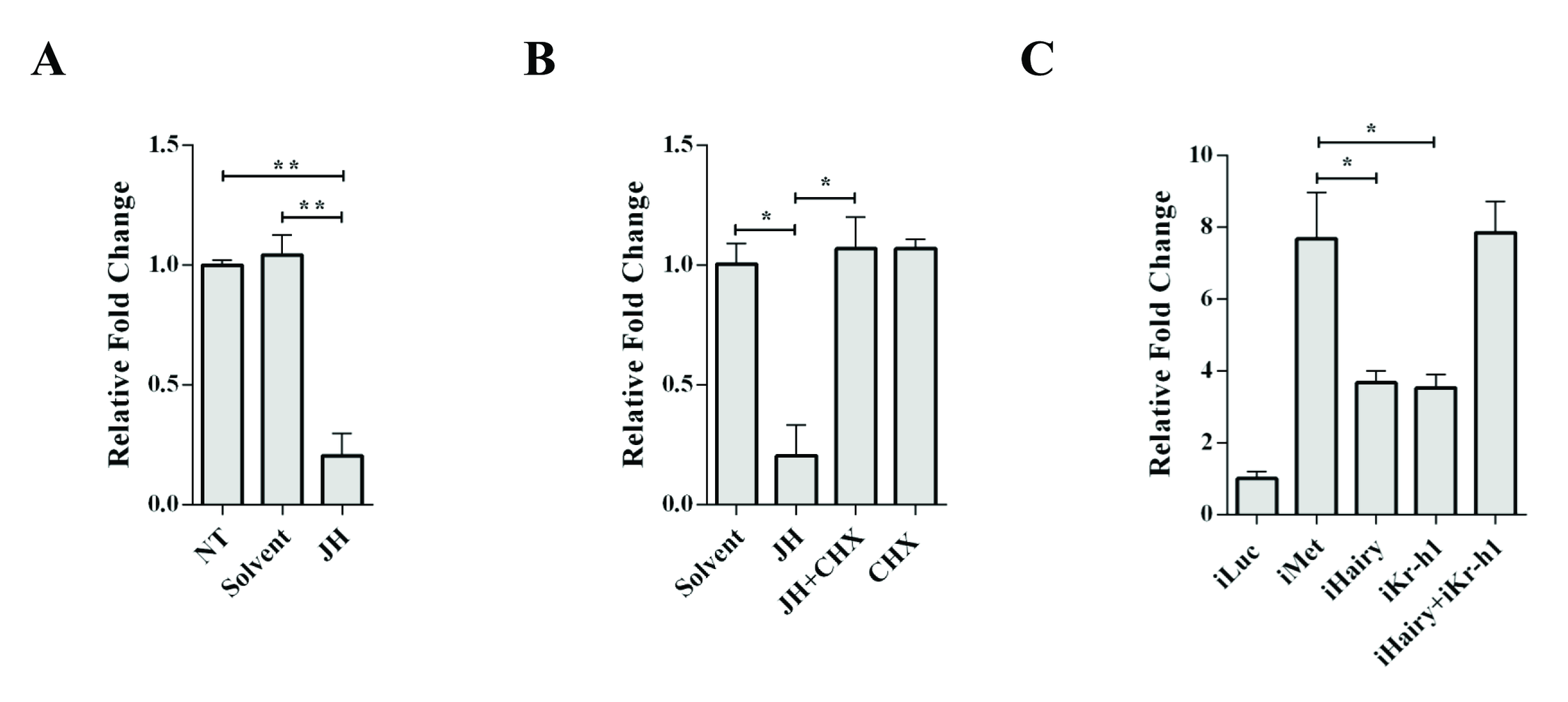

Supplement: S2 Fig — (A) Hormonal application experiments showing the effect of JH on the expression of AAEL006978 (protein-glutamine gamma-glutamyltransferase). A clear repression of the tested gene was observed in JH-treated samples. NT, no treatment. (B) The necessity of intermediate factors for the JH-mediated repression of the target gene AAEL006978 as determined by in-vitro fat body culture experiments. JH-mediated repression was compromised by the addition of CHX into the tissue culture medium (JH+CHX). Solvent (acetone) and CHX-treated samples were used as controls. (C) qRT-PCR-based expression analysis of Met/Hairy/Kr-h1 target gene AAEL006978 in iMet, iHairy, iKr-h1 and iHairy+iKr-h1 samples. iLuc samples were used as controls. Error bars represent ± SD. *p < 0.05; ***p < 0.001. (TIF) [file pgen.1008443.s002.tif]

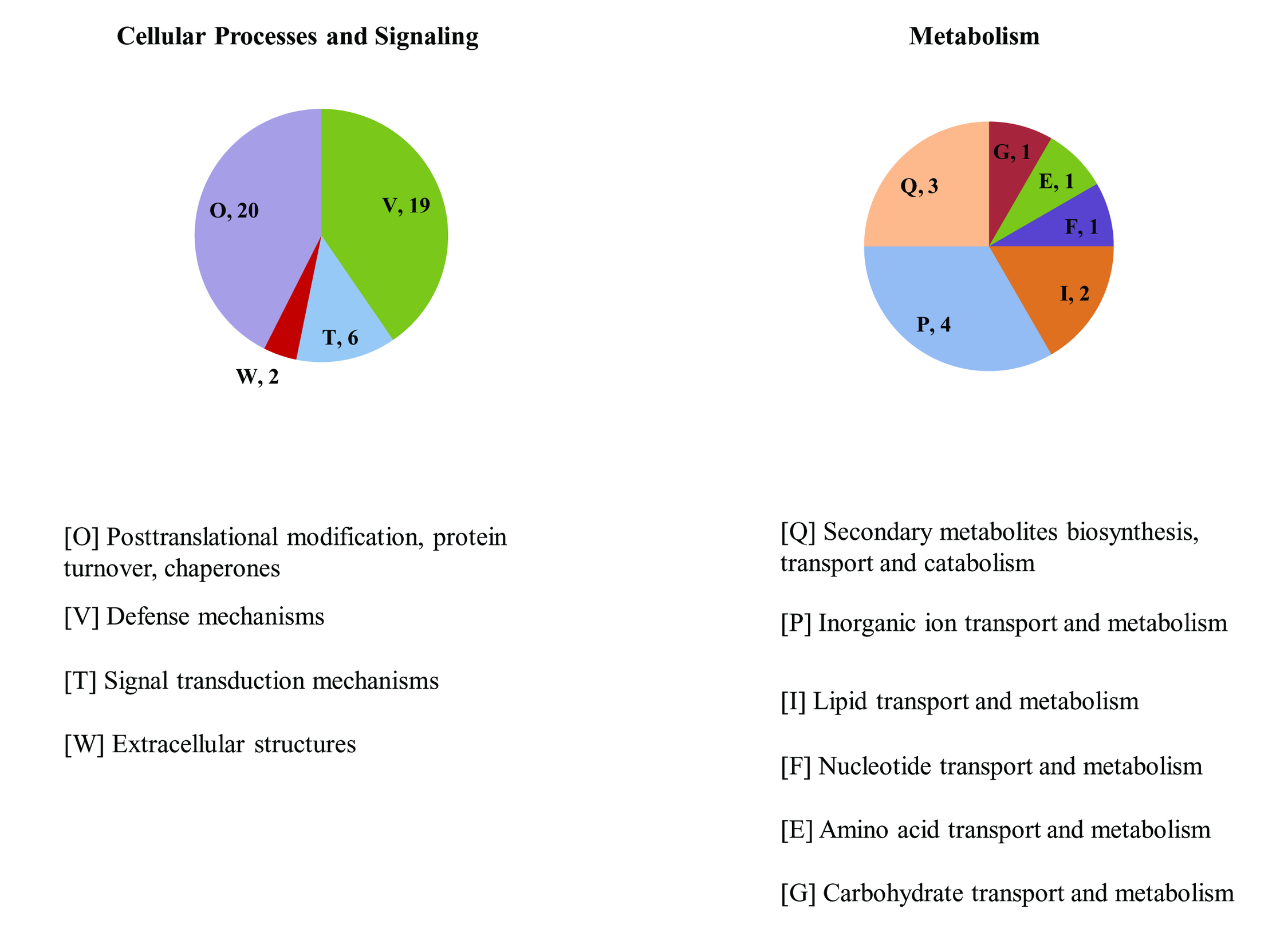

Supplement: S3 Fig — All 130 transcripts activated in the fat body by dsRNA-mediated knockdown of Met, Hairy, and Kr-h1 were mapped to get an overview of the functional groups affected by the transcription factors. Two major categories Cellular Processes and Signaling (left) and Metabolism (right) are shown as pie diagrams. Functional groups with corresponding abbreviations and colors are indicated. The other major category- Information Storage and Processing (not shown in the graph) was not represented in the iMet/iHairy/iKr-h1-activated gene set, with no transcript mapped to the subcategories under this heading. (TIF) [file pgen.1008443.s003.tif]

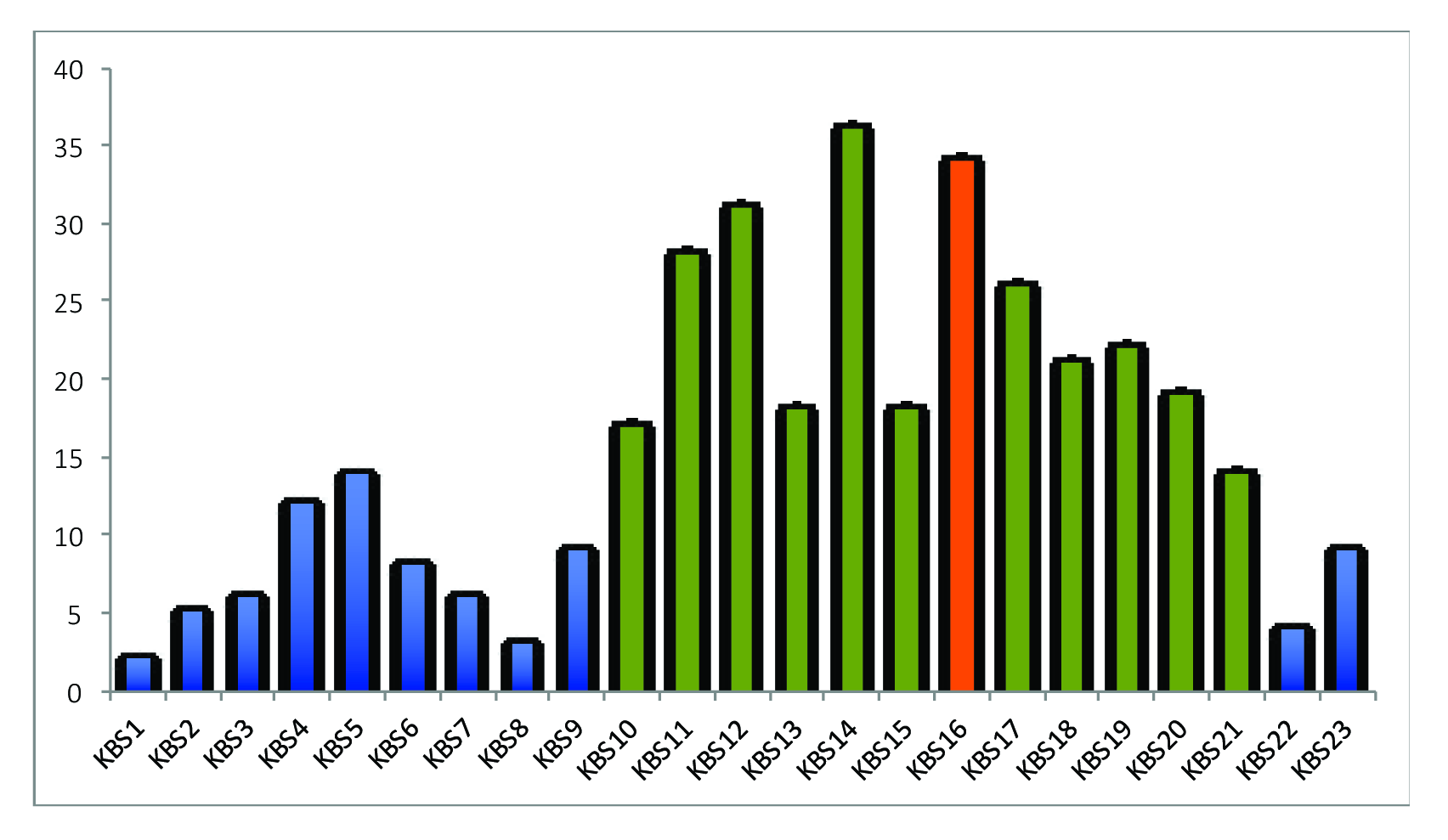

Supplement: S4 Fig — The graph showing the frequency of hits for all the 8-mer sequences generated for the characterization of potential Kr-h1 binding site (KBS) (from Fig 5A) in the region 5-kb upstream of iMet/iKr-h1/iHairy upregulated transcripts. The 23 8-mers used are listed in Fig 5A. (TIF) [file pgen.1008443.s004.tif]

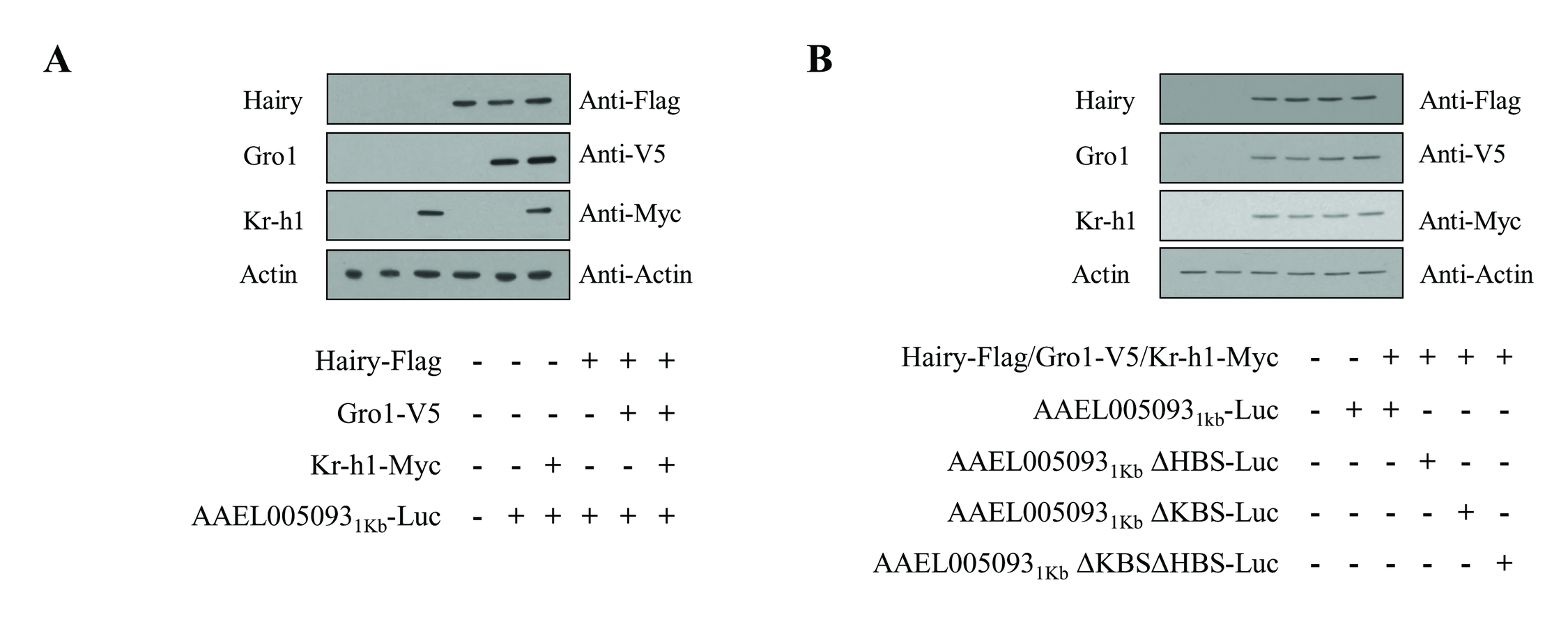

Supplement: S5 Fig — (A and B) Western blots showing the expression of tagged proteins Kr-h1-Myc, Hairy-Flag and Gro1-V5 in cell culture samples in Fig 6A and 6B, respectively. Commercially available anti-Myc, Flag and V5 antibodies were utilized for the Western blot analysis. Actin was used as a loading control. (TIF) [file pgen.1008443.s005.tif]

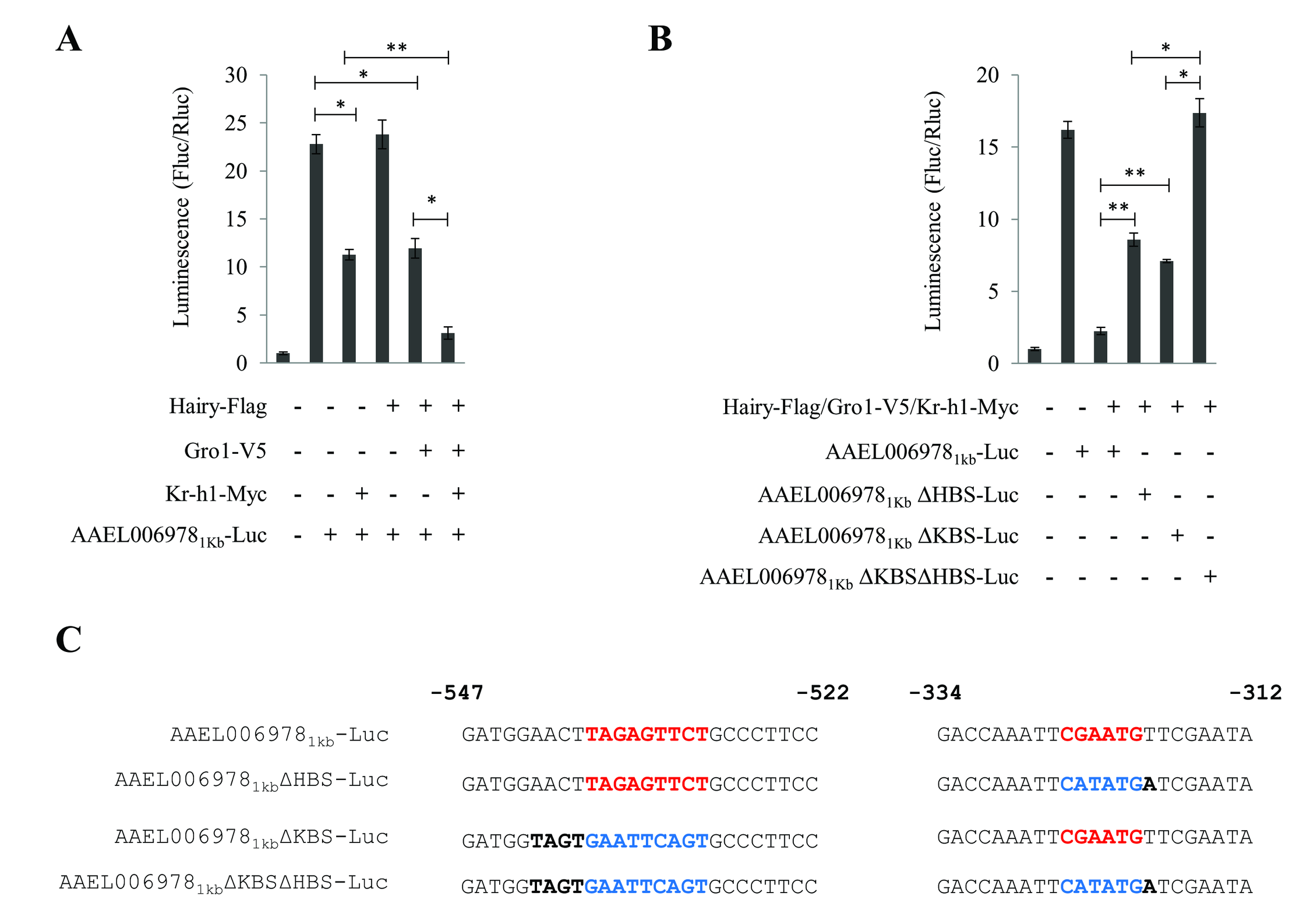

Supplement: S6 Fig — (A) Luciferase reporter assays after co-transfection of expression vectors Hairy-Flag and/or Gro1-V5 and/or Kr-h1-Myc along with reporter construct AAEL0069781kb-Luc. Treatments with no input DNA and empty expression vector served as controls. Repression of promoter activity was observed when either Kr-h1-Myc or Hairy-Flag was overexpressed in the cell culture system. Co-repressor Gro1-V5 was required for the successful functioning of Hairy. However, overexpression of both Kr-h1-Myc and Hairy-Flag (along with Gro1-V5) dramatically enhanced the intensity of repression of luciferase activity. Error bars represent ± SD. *p < 0.05; ***p < 0.001. (B) Mutation analysis of AAEL006978 promoter by luciferase reporter assays. The Kr-h1 and Hairy binding sites (KBS and HBS, respectively) in the AAEL0069781kb-Luc reporter construct were mutated either separately (AAEL0069781kb ΔHBS-Luc and AAEL0069781kb ΔKBS-Luc) or together (AAEL0069781kb ΔKBSΔHBS-Luc) and co-transfected along with expression vectors Hairy-Flag and Gro1-V5 and Kr-h1-Myc. The repression in the luciferase activity observed with the AAEL0069781kb-Luc construct was partially compromised with the mutation of either KBS or HBS. A complete loss of repression in the promoter activity was observed when both KBS and HBS were mutated in the promoter upstream of the luciferase gene in reporter construct. Error bars represent ± SD. *p < 0.05; ***p < 0.001. (C) Predicted KBS and HBS along with their flanking regions harbored within 1kb of the AAEL006978 promoter and the various promoter mutations utilized in (A) and (B) are indicated. (TIF) [file pgen.1008443.s006.tif]
